# Oligotyping reveals stronger relationship of organic soil bacterial community structure with N-amendments and soil chemistry in comparison to that of mineral soil at Harvard Forest, MA, USA

**DOI:** 10.3389/fmicb.2015.00049

**Published:** 2015-02-16

**Authors:** Swathi A. Turlapati, Rakesh Minocha, Stephanie Long, Jordan Ramsdell, Subhash C. Minocha

**Affiliations:** ^1^Department of Biological Sciences, University of New HampshireDurham, NH, USA; ^2^Northern Research Station, United States Department of Agriculture Forest ServiceDurham, NH, USA; ^3^Hubbard Center for Genome Studies, University of New HampshireDurham, NH, USA

**Keywords:** bacterial community, forest soils, microbiome, oligotypes, pyrosequencing, QIIME software, OTUs, entropy

## Abstract

The impact of chronic nitrogen amendments on bacterial communities was evaluated at Harvard Forest, Petersham, MA, USA. Thirty soil samples (3 treatments × 2 soil horizons × 5 subplots) were collected in 2009 from untreated (control), low nitrogen-amended (LN; 50 kg NH_4_NO_3_ ha^-1^ yr^-1^) and high nitrogen-amended (HN; 150 kg NH_4_NO_3_ ha^-1^ yr^-1^) plots. PCR-amplified partial 16S rRNA gene sequences made from soil DNA were subjected to pyrosequencing (Turlapati et al., 2013) and analyses using oligotyping. The parameters M (the minimum count of the most abundant unique sequence in an oligotype) and s (the minimum number of samples in which an oligotype is expected to be present) had to be optimized for forest soils because of high diversity and the presence of rare organisms. Comparative analyses of the pyrosequencing data by oligotyping and operational taxonomic unit clustering tools indicated that the former yields more refined units of taxonomy with sequence similarity of ≥99.5%. Sequences affiliated with four new phyla and 73 genera were identified in the present study as compared to 27 genera reported earlier from the same data (Turlapati et al., 2013). Significant rearrangements in the bacterial community structure were observed with N-amendments revealing the presence of additional genera in N-amended plots with the absence of some that were present in the control plots. Permutational MANOVA analyses indicated significant variation associated with soil horizon and N treatment for a majority of the phyla. In most cases soil horizon partitioned more variation relative to treatment and treatment effects were more evident for the organic (Org) horizon. Mantel test results for Org soil showed significant positive correlations between bacterial communities and most soil parameters including NH_4_ and NO_3_. In mineral soil, correlations were seen only with pH, NH_4_, and NO_3_. Regardless of the pipeline used, a major hindrance for such a study remains to be the lack of reference databases for forest soils.

## INTRODUCTION

Soils harbor an immense diversity of bacteria (Torsvik et al., 2002; Trevors, 2010 and references therein), most of it is hidden from experimental analyses (Wall et al., 2010). A vast majority of soil microbes are recalcitrant to culture methods thus increasing the complexity of any study to expose this concealed diversity (Sait et al., 2002; Nunes da Rocha et al., 2009; Vartoukian et al., 2010; Lombard et al., 2011). Recently developed molecular tools have enabled us to analyze the expanse of variation in bacterial populations using culture-independent methods such as polymerase chain reaction (PCR) amplification of partial or full-length genes of 16S rRNA, and their in-depth sequencing (Janssen, 2006; Větrovský and Baldrian, 2013). Next generation sequencing approaches (e.g., pyrosequencing and Illumina technology) generate data that are several orders of magnitude superior than traditional sequencing methods; still they have limited ability for the assessment of the total microbial diversity in a soil sample, albeit the taxonomic richness (Margulies et al., 2005; Huse et al., 2009; Gloor et al., 2010). This is primarily due to the lack of reference genome libraries even for the dominant bacterial species in forest soil ecosystems (Howe et al., 2014).

In nature, microbes are vital contributors to biogeochemical transformations and hence examining their response to anthropomorphic activities over long periods is critical for understanding ecological processes (Falkowski et al., 2008). Several studies have been conducted to reveal the extent of diversity of the soil bacterial and fungal communities being influenced by various factors including aboveground plant populations (Carney and Matson, 2006; Uroz et al., 2010; McGuire et al., 2012), soil type (Roesch et al., 2007; Lauber et al., 2008), soil pH (Fierer and Jackson, 2006; Lauber et al., 2009; Rousk et al., 2010), and differences in geographic location (Fulthorpe et al., 2008; Langenfeld et al., 2013; Bischoff et al., 2014). It is evident that soil microbial communities are influenced by numerous human activities, particularly land management practices, including long term nitrogen (N) fertilization of both agricultural and forest soils (Wu et al., 2008, 2011; Hallin et al., 2009; Ramirez et al., 2010; Fierer et al., 2012; Sridevi et al., 2012; Coolon et al., 2013; Turlapati et al., 2013). Wagg et al. (2014) have recently stressed the importance of changes in soil microbial diversity on nutrient cycling at several sites.

During the 1990s, N added to the atmosphere through human activity was much higher (160 Tg Y^-1^) than through natural biological fixation processes (110 Tg Y^-1^) (Gruber and Galloway, 2008). These authors suggested that increased N has multiple effects on cycling of other elements in the environment including carbon (C), leading to global warming. Acidification due to N saturation causes forest soils to become deficient in important labile pools of nutrients, particularly Ca^2+^ and Mg^2+^ (Currie et al., 1999). Calcium deficiency in the soil is known to predispose plants to disease and pathogen infection, thus contributing to decline in forest productivity as seen in red spruce and sugar maple stands in the Northeastern US (Shortle and Smith, 1988; Long et al., 1997; Minocha et al., 2010; Schaberg et al., 2011). These aboveground changes are often accompanied by belowground changes in soil chemistry that alter the microbiome, which in turn impacts the biogeochemical cycling of essential nutrients (N, C, and P). Recent studies have reported reduced microbial biomass and activity with N fertilization of forest soils (Treseder, 2008; Janssens et al., 2010). Other reports have indicated either an increase (Cusack et al., 2010), or a neutral response (Zhao et al., 2013) in microbial biomass with N fertilization. The variability of these findings suggests that the effects of N addition on microbial populations may be site-specific.

At the HF Long-Term Ecological Research site located in Petersham, MA, USA (HF)^1^, experimental plots were set up in 1989 to study the long-term effects of N addition on above- and belowground communities (Magill et al., 2004). Past studies from this site have shown negative shifts in the ratio of fungal: bacterial biomass, microbial biomass C, and substrate-induced respiration rates in response to N additions (Bowden et al., 2004; Frey et al., 2004; Wallenstein et al., 2006; Ramirez et al., 2012). Soil from N treatment plots at HF were reported to accumulate more C due to a decrease in decomposition rate (Frey et al., 2014). Restriction fragment length polymorphism (RFLP) and PCR profiles for DNA extracted from N-treated soil samples exhibited altered functional N-cycle gene composition (Compton et al., 2004). Using pyrosequencing of the PCR-amplified 16S rRNA genes from the soil DNA, our group showed that N addition caused profound rearrangements in the structure of bacterial communities at the HF site (Turlapati et al., 2013). Major changes were recorded in the community structure of *Acidobacteria*, α and β subclasses of *Proteobacteria, and Verrucomicrobia* were observed. These conclusions were derived using the UCLUST tool in Quantitative Insights into Microbial Ecology (QIIME) toolkit using the latest version available (1.4.0) at that time (Caporaso et al., 2010b; Edgar, 2010) to cluster the sequences into operational taxonomic units (OTUs) at 97% sequence identity. It was observed that 2% of the total OTUs in this dataset contained ≥50% of the total sequences; on the other hand, up to 80% of total OTUs were highly diverse and contained ∼10% of the total sequences.

It has been suggested that there is substantial phylogenetic diversity in marine and soil environments attributable to the occurrence of rare bacterial populations (Sogin et al., 2006; Lynch et al., 2012). Although the diversity of HF soil microbes could be estimated to some extent in our previous study, it was not possible to examine the sequence diversity within each abundant OTU since classification was assigned only to the OTU representative sequences (Turlapati et al., 2013). This is important because OTU clustering (often done at 97% sequence identity) is less powerful in identifying phylogenetically distinct organisms that differ by a small number of nucleotides (Eren et al., 2013). Oligotyping is a recently developed computational tool that allows users to choose entropy components (‘supervised tool’) that have high variability in order to resolve underlying diversity among sequences within each OTU or taxonomic group (Eren et al., 2013).

With the aim of analyzing the effects of prolonged N treatment on individual bacterial groups, and identifying additional genera/families whose presence and/or abundance may be correlated with alterations in soil factors, we subjected our pyrosequencing dataset (Turlapati et al., 2013) to the oligotyping pipeline and taxonomy was assigned using the recently updated RDP database (Cole et al., 2013). The specific objectives of the study were to: (1) demonstrate the applicability of the oligotyping pipeline for forest soil datasets; (2) study the effects of N-amendment on individual bacterial taxa and compare these with previous findings based on OTU clustering; and (3) evaluate the effects of soil chemistry on bacterial communities of Org and Min horizons.

## MATERIALS AND METHODS

### SITE DESCRIPTION AND SOIL SAMPLE COLLECTION

The study site is a mixed hardwood stand naturally regenerated after being clear-cut in 1945 and is located on Prospect Hill at the HF^2^, Petersham, MA, USA. The stand is comprised of predominantly red oak (*Quercus rubra L*) and black oak *(Q. vetulina* Lam.), mixed with red maple (*Acer rubrum* L.), American beech (*Fagus grandifolia* Ehrh.) and black birch (*Betula lenta* L.). Soil at this site is mostly stony to sandy loam formed from glacial till. For more details on site description including vegetation, climate, site topography, and N amendments refer to Aber et al. (1993) and Magill et al. (2004).

As described in our previous report (Turlapati et al., 2013), three 30 m × 30 m treatment plots (further subdivided into 36 subplots; each measuring 5 m × 5 m) were used for sample collection. These plots were established in May 1988 as part of a long-term study on chronic N effects on ecosystem function. One plot served as a Con and 2 other plots were treated with NH_4_NO_3_; low N (LN; treated with 50 kg N ha^-1^ yr^-1^), and high N (HN; treated with 150 kg N ha^-1^ yr^-1^). Ammonium nitrate solution was applied by a backpack sprayer yearly in six equal doses at 4-week intervals from May to September. Soil samples were collected from five randomly selected subplots within each treatment plot in September 2009 using a soil corer (7.5 cm diameter). The upper Org layer (Org, average 8 cm) was separated from the lower Min layer. In a few cases where the Org horizon was 10–12 cm deep, deeper coring was needed to get to the Min soil. Thirty samples (5 cores per plot × 2 horizons × 3 treatments) were collected in polyethylene bags and brought to the laboratory on ice. Samples were sieved (2 mm pore size) to remove roots, debris and stones, and then stored at -20^∘^C for further use.

### SOIL CHEMICAL ANALYSES

Air-dried soil samples (20–40 g) were sent to the Soil Testing Service Laboratory at the University of Maine, Orono, ME, USA^3^ for analyses. Nitrate and NH_4_ -N were extracted in potassium chloride and determined colorimetrically by Ion Analyzer in 2012. The rest of the analyses were carried out in 2010 as described in Turlapati et al. (2013). The methods for the extraction of polyamines and amino acids were described in our previous publication (Frey et al., 2014).

### DNA ISOLATION, PCR, PYROSEQUENCING, AND DATA QUALITY FILTERING

As previously described in Turlapati et al. (2013), PowerSoil®; DNA isolation kit (MO-BIO Laboratories, Carlsbad, CA, USA) was used to isolate genomic DNA from 0.5 g of soil samples. Universal primers (F968 5^′^AA CGC GAA GAACCT TAC3^′^ and R1401-1a 5^′^CGG TGT GTA CAA GGC CCG GGA ACG3^′^) as described in Brons and van Elsas (2008) with 30 barcodes (10 bp, one for each soil sample) were used for PCR to generate ∼433-bp amplicons corresponding to the V6–V8 hypervariable region of the bacterial 16S rRNA encoding gene. The PCR amplifications were conducted in triplicate using Phusion®; Taq Master Mix (New England Biolabs, Ipswich, MA, USA) with 50 ng of template DNA in a final volume of 50 μL. The reactions were performed in a PTC-100®; Programmable Thermal Cycler (MJ Research, Inc., Waltham, MA, USA) with the following conditions: an initial denaturation at 95^∘^C for 5 min, followed by 20 cycles of denaturation at 95^∘^C for 30 s, annealing at 61^∘^C for 30 s, and extension at 72^∘^C for 45 s, with a final extension at 72^∘^C for 10 min. The triplicate reaction products (amplicons) from each soil sample were pooled for sequencing. DNA purification kit (Zymo Research, Irvine, CA, USA) was used to purify the pooled PCR products which were then subjected to further cleaning via the Agencourt®; AMPure®; XP Bead Purification method (Agencourt Bioscience Corporation, Beverly, MA, USA) to remove fragments <100 bp. The quality of the PCR products were evaluated in an Agilent 2100 Bioanalyzer using the DNA 1000 LabChip (Agilent Technologies, Palo Alto, CA, USA). The 30 bar-coded samples were pooled in equimolar quantities (Margulies et al., 2005) in order to process for sequencing (Roche 454 GS-FLX Titanium System) at the University of Illinois, USA^4^ in a full picotiter plate.

### OLIGOTYPING ANALYSIS

The forward primer (549,500 sequences) pyrosequencing data were quality filtered in QIIME (version 1.4.0) with default settings for most steps as described in Caporaso et al. (2010b). Chimeric sequences were also removed using Chimera Slayer in QIIME (Table S1). After removal of low quality and chimeric sequences, the remaining data were used as an input taxonomic assignment using the Ribosome Database Project (RDP) classifier version 2.7^5^. In the first step, IDs of the sequences corresponding to each phylum were extracted individually from the dataset using CT ≥0.8 with Perl script (Supplementary Material – 1). The sequences corresponding only to these IDs (selected at CT ≥0.8) were then extracted out by using the command filter.fasta.py (available in QIIME) and were further processed for oligotyping analyses. Selected sequences corresponding to a phylum or a subgroup/class were aligned using PyNAST (Caporaso et al., 2010a). PyNAST enables the alignment of sequences against a template database such as Greengenes (McDonald et al., 2012) in QIIME. Before beginning oligotyping, the uninformative gaps in the alignment were removed along with 10–15 nucleotides at the end of each read, which were trimmed to attain reads of similar lengths.

Oligotyping was conducted individually for each phylum. The only exceptions were *Proteobacteria* and *Acidobacteria* where oligotyping had to be conducted individually for each class or subgroup because among some of the subgroups there were several-fold differences in the number of sequences (e.g., within *Acidobacteria*, subgroup *Gp*1 accounted for 151,462 sequences and *Gp*4 had only 715 sequences); these required different *M* values (the minimum substantive abundance of the most abundant unique sequence in an oligotype) for analyses. To begin with all sequences were 430 bp in size; after alignment and filtering the gaps, the sizes of the aligned reads were different for different phyla (Figure S1).

Entropy and initial oligotyping analyses were conducted according to Eren et al. (2013). The oligotyping method utilizes Shannon (1948) entropy for detecting the amount of diversity associated with each nucleotide position and provides a way of identifying positions associated with greater variability. The entropy peaks for nucleotide positions ranged from 0 to slightly >2 for most of the datasets under consideration with the exception of the phylum *Nitrospira* (Figure S1). We observed that a higher number of peaks with entropy values ≥1.0 resulted in greater bacterial diversity. In the first step, oligotypes were generated by using the first position with the highest entropy value. To decompose these oligotypes further, supervised oligotyping steps (user-defined nucleotide selection for decomposing entropy) were used. On the average 50 positions were chosen to decompose entropy for most datasets. Oligotyping was continued until no peaks were left unresolved that could further decompose the oligotype. Peaks with entropy values of <0.2 often did not yield additional oligotypes and were considered background noise. Details for the above steps are described in Eren et al. (2013).

For supervised oligotyping, *M* values had to be modified for forest soil samples from those suggested for other ecosystems by Eren et al. (2013). Since the total number of sequences varied across different bacterial phyla, the *M* values varied accordingly for each analysis as shown in Table S2. This value was usually lower (2–5) for relatively smaller datasets in the range of 200–5000 reads. In order to correct for technical errors due to pyrosequencing, the value for parameter ‘s’ (‘s’ is the minimum number of samples in which an oligotype is expected to be present) was set to two with the assumption that any sequence that occurs in two biological samples represents an element of a microbiome and is not an error. Huse et al. (2010) found that even with deep sequencing, OTUs with one sequence rarely occurred in other replicates and the chance of a spurious OTU occurring in two environmental samples is not realistic which supports our assumption. To capture the rare biosphere in soil samples, no values were assigned to parameter ‘a’ (the minimum percent abundance of an oligotype in at least one sample) and parameter ‘A’ (the minimum actual abundance of an oligotype across all samples). Oligotype representative sequences (the most abundance sequence within an oligotype) were classified at CT ≥0.8 (a generally accepted threshold of 80% for assigning taxonomy) using the RDP classifier tool version 2.7^6^. For determining percent alignment scores, the sequences corresponding to individual oligotypes were extracted from the oligo-representatives directory and aligned using the ClustalW2 tool^7^.

In one small comparative study with a subset of data, oligotype representative sequences were classified at CT ≥0.5 as well as ≥0.8 using the RDP classifier to determine if additional genera could be identified using a lower CT value.

The oligotype representative sequences have been deposited in the NCBI short read archive. The accession numbers are presented in Table S1.

### OTU CLUSTERING AND TAXONOMIC ANALYSES

To compare the oligotyping method with OTU clustering, quality-filtered reads of four phyla (*Actinobacteria, Bacteroidetes, Firmicutes,* and *Proteobacteria*) were selected and clustered into OTUs using UCLUST (Edgar, 2010) set at a 97% identity threshold in QIIME (version 1.8.0). The OTU representative sequences were picked in QIIME based on the most abundant sequence in each OTU. Similarly, the oligotyping method also assumes that the most abundant unique sequence is the oligotype representative sequence. For each dataset, sequences were filtered for minimum abundance (*n* size) for each OTU using the same value that was used for *M* in the corresponding oligotyping analyses. In addition, in order to match the parameter set for oligotyping (*s* = 2), OTUs that were not present in at least two samples were removed from the OTU table using python scripts (Supplementary Material – 2). The OTU representative sequences were classified using RDP version 2.2 (currently used version) in QIIME.

In order to understand the reason for the difference in the genera identified by the two methods, we assigned taxonomy to OTU and oligo representative sequences using RDP in QIIME pipeline version 2.2 and online RDP classifier version 2.2.

### STATISTICAL ANALYSES

SYSTAT (version 10.2, Systat Software, Inc., San Jose, CA, USA) was used for standard statistical tests, including paired *t*-tests and two-way analysis of variance (ANOVA), on the soil NH_4_ and NO_3_ data. Non-metric dimensional scaling (NMS) analyses were conducted using PC-ORD (version 6.03, MJM Software Design, Gleneden Beach, OR, USA). To normalize the data, digit one was added to all data before log10 transformation. Briefly, following settings were used for NMS: number of axes = 4, maximum number of iterations = 500; stability criterion (the standard deviation in stress over the last 10 iterations) = 10^-6^; number of runs with real data = 100; and the number of runs with randomized data = 250. Random numbers were chosen as a source of starting ordinations. The tie handling was done by penalizing unequal ordination distance (Kruskal’s secondary approach). The following were chosen as output options: varimax, randomization test, plot stress vs. iterations and calculate scores for OTUs by weighted averages. Dimensionality of solutions was selected for these analyses based on the assessment using a graph of stress as a function of dimensionality (scree plot). A Monte Carlo test was used to examine the stress and the strength of NMS results. Two-way permutational MANOVA was conducted using the Bray-Curtis distances to evaluate the effect of the horizons and the treatment, and the interaction between them. Mantel tests were conducted to evaluate the significance of correlations among Bray-Curtis distance scores and soil chemistry and soil Org N metabolites.

## RESULTS

### SOIL CHEMISTRY

While NH_4_ concentration in the Org soil was significantly higher than that in the Min soil for all treatments (Table S3), there was no difference in NO_3_ levels between the two horizons. Long-term N treatment did not significantly alter either NH_4_ or NO_3_ concentrations of either soil horizon (Table S3). Other details on soil analyses are described in Frey et al. (2004) and Turlapati et al. (2013).

### PARAMETERS OF OLIGOTYPING ANALYSES

Soils are highly diverse and harbor an abundance of rare microbes. Rare microbes are more prone to primer-PCR amplification and sequencing biases thus making it harder to identify such individuals. In addition, it is well known that soil replicates have high microsite variability in chemistry as well as bacterial populations. This combination of rarity and microsite variability perhaps is the reason that the same microbes are not present in all replicate samples, and why the guidelines suggested for other biomes in Eren et al. (2013) did not work with the HF soil samples. Thus for analysis of these soils, the suggested guidelines had to be modified (personal communication with Dr. A. Murat Eren, Marine Biological Laboratories, Woods Hole, MA, USA).

*M* is the value that is used to filter out potential noise in a sample. For this reason it is generally kept at a reasonably high number. It is likely that with forest soils, sequences representing rare taxa may be filtered out as noise due to their low abundance at high *M* values. In addition, high *M* values in such cases filtered out more than 50% of all sequences. The more diverse the phyla are (in terms of number of entropy components) more are the sequences that are filtered out with high *M* values (Table S2, e.g., see *Bacteroidetes vs. Gp10*). With the goal of retaining maximum sequences and diversity in terms of the number of oligotypes, several *M* values were tested for most phyla before final analyses. We tested different *M* values *using* α*-* and β*-Proteobacteria*, two well-known bacterial classes that are highly diverse and varied in the size of data for our soil samples with 38,858 and 3,340 sequences, respectively. The *s* value was set at two for both analyses. Lowering *M* values resulted in the identification of more genera at CT ≥0.8 in both classes (**Table 1**). For example at CT of ≥0.8, the total number of genera identified with an *M* = 75 for α*-Proteobacteria* was 5, but with *M* = 15, 11 genera were identified. Similarly, for β*-Proteobacteria, M* = 25 identified only two genera while *M* = 3 identified eight genera (**Table 1**) In α*-Proteobacteria*, genera such as *Labrys* and *Acidisoma* were identified with *M* = 15; they would be missed at higher *M* values. Similar results were obtained within β*-Proteobacteria* (**Table 1**). Therefore, final data analyses in this study were conducted using an *M* value that was based on two criteria, namely, the retention of maximum sequences, and maximum diversity (in terms of number of oligotypes) with taxonomic assignment at CT values no less than 0.8. Data presented here show that using high *M* values for groups that have low abundance would not identify genera with high confidence limits [e.g., at *M* = 2 *Paenibacillus* (*Firmicutes*) at CT = 1 and *Mucilaginibacter* (*Bacteroidetes*) at CT of 0.99–1 were identified].

**Table 1:** Effect of varying *M* values on percent of reads retained, total number of oligotypes, and genera identified at 0.8 CT with RDP database.

Class	*M* value	% of reads retained	Number of oligotypes	Genera identified at 0.8 CT in RDP	Total number of genera
α*-Proteobacteria*	75	35	73	*Bradyrhizobium, Rhodomicrobium, Rhizomicrobium, Methylocella, Methylosinus*	5
”	50	44	118	*Bradyrhizobium, Rhodomicrobium, Rhizomicrobium, Methylocella, Methylosinus, Hyphomicrobium, Phenylobacterium*	7
”	30	55	211	*Bradyrhizobium, Rhodomicrobium, Rhizomicrobium, Methylocella, Methylosinus, Hyphomicrobium, Phenylobacterium, Acidisphaera, Bauldia*	9
”	15*^#^	67	389	*Bradyrhizobium, Rhodomicrobium, Rhizomicrobium, Methylocella, Methylosinus, Hyphomicrobium, Phenylobacterium, Acidisphaera, Bauldia, Labrys, Acidisoma*	11
*β-Proteobacteria*	25	64	27	*Burkholderia, Herbaspirillum*	2
”	20	65	29	*Burkholderia, Herbaspirillum*	2
”	15	69	37	*Burkholderia, Herbaspirillum, Comamonas, Herminiimonas*	4
”	10	74	48	*Burkholderia, Herbaspirillum, Comamonas, Herminiimonas, Collimonas*	5
”	5^$^	81	83	*Burkholderia, Herbaspirillum, Comamonas, Herminiimonas, Collimonas, Nitrosospira, Variovorax*	7
”	3*^$^	86	123	*Burkholderia, Herbaspirillum, Comamonas, Herminiimonas, Collimonas, Nitrosospira, Variovorax, Aquabacterium*	8
”	2	88	157	*Burkholderia, Herbaspirillum, Comamonas, Herminiimonas, Collimonas, Nitrosospira, Variovorax, Aquabacterium*	8

The datasets used for illustrating this point are α*-Proteobacteria* (38,858 sequences) and β*-Proteobacteria* (3,440 sequences); s value used for these analyses was 2. *Indicates final *M* values used for the rest of the analyses. ^#^Indicates that below an *M* value of 15 a machine generated error was encountered due to exceedingly large numbers of oligotypes. ^$^Indicates that *Nitrosospira, Variovorax*, and *Aquabacterium* were identified at CT of (0.98–1).

### ALIGNMENT OF SEQUENCES WITHIN OLIGOTYPES

We compared the sequence identities within OTUs clustered at ≥97% similarity (Edgar, 2010) in QIIME with those in oligotypes that are generated by manually selecting components of high entropy values. The results show that the sequence identities within an oligotype ranged between 99.5 and 100%. In order to reduce variation among sequence identities within an oligotype, all peaks with entropy values greater than 0.6 were resolved. The oligotyping process left very few unresolved peaks of relatively low entropy (<0.2) values within oligotypes that often had <100 sequences. These low entropy peaks were considered as the background noise (**Table 2**). However, even when the oligotyping analysis appeared not fully resolved, the range of % identities was still within 99.76–100%. **Table 2** shows two such examples: in one case there are two peaks with entropy values of <0.25, and in another case there is only one large peak with an entropy value at 0.65. In such cases, the small peaks were deliberately disregarded because their further decomposition did not result in additional oligotypes. The sequence identities within OTUs clustered using the same dataset ranged from 97 to 100% (results not shown).

**Table 2:** CLUSTALW percent identity alignment scores for the sequences within each oligotype, taxonomic affiliation of the oligotype, total number of unresolved peaks, and the entropy values associated with the nucleotide components of unresolved peaks.

Oligo ID	Taxonomic affiliation	Number of sequences within oligotype (number of 100% identical sequences)	% identity among the sequences	Number of unresolved peaks	Entropy associated with the nucleotide components of unresolved peaks
00054	*Acidobacteria: Gp1*	498 (462)	99.53–100	Background noise*	<0.10
00009	*Acidobacteria: Gp3*	469 (436)	99.30–100	Background noise*	<0.15
00008	*α-Proteobacteria*	276 (255)	99.53–100	Background noise*	<0.10
00012	β*-Proteobacteria*	52	100	none	–
00028	β*-Proteobacteria*	23 (22)	99.76–100	2	0.25
00040	β*-Proteobacteria*	14 (13)	99.76–100	2	0.37
00165	*Verrucomicrobia*	54 (43)	99.54–100	1	0.65

*Background noise is defined as the situation when the unresolved peaks with entropy values associated with the nucleotide components fall within the range of 0.1–0.2. This often happened with oligotypes having >100 sequences.

### TAXONOMIC ASSIGNMENTS USING DIFFERENT TOOLS

Major motivation for examining the use of oligotyping as a tool was to reveal concealed microbial population diversity that could not be seen using the OTU clustering approach (Turlapati et al., 2013) with the additional focus on the effects of N treatment on the distribution of these taxa. Comparison of taxonomic assignments between OTUs described earlier using QIIME 1.4.2 and the data presented in this report show more genera were identified using the oligotyping analysis (**Table 3**). Although the same data set was used for both analyses, genus level information for phyla such as *Acidobacteria*, *Bacteroidetes,* and *Chloroflexi* was generated in the present study. It should be pointed out that the use of an updated version (2.7) of RDP also revealed four previously unidentified phyla (AD3*, Cyanobacteria,* TM6 *and* WPS-2; Table S2).

**Table 3:** Comparison of genera identified by QIIME (version 1.4.0) UCLUST clustering [method used in our previous study (Turlapati et al., 2013)] with those identified in the present study using oligotyping.

Phylum/class	Genera identified in Turlapati et al. (2013) using QIIME UCLUST clustering method	Total number	Genera identified in present study using oligotyping	Total number
*Acidobacteria*	–	0	*Acidobacterium, Bryobacter, Edaphobacter, Granulicella*	4
α*-Proteo*	*Bradyrhizobium, Methylocella, Phenylobacterium*, *Rhodomicrobium*	4	*Acidisoma, Acidisphaera, Bauldia, Bradyrhizobium, Hyphomicrobium, Labrys, Methylocella, Methylosinus, Phenylobacterium, Rhizomicrobium, Rhodomicrobium*	11
β*-Proteo*	*Burkholderia*	1	*Aquabacterium, Burkholderia, Collimonas, Comamonas, Herbaspirillum, Herminiimonas, Nitrosospira, Variovorax*	8
δ*-Proteo*	Byssovorax*	1	*–*	0
γ*-Proteo*	*Aquicella, Dyella, Legionella, Pseudomonas, Serratia*, *Stenotrophomonas*	6	*Aquicella, Coxiella, Dyella, Legionella, Nevskia, Pseudomonas, Rhodanobacter, Rudaea, Serratia, Stenotrophomonas, Yersinia*	11
*Actinobacteria*	Actinospica, Actinocorallia*, Catenulispora, Conexibacter, Kitasatospora, Mycobacterium, Nocardia,	7	*Aciditerrimonas, Actinospica, Catenulispora, Conexibacter, Kitasatospora, Microbacterium, Mycetocola, Mycobacterium, Nocardia, Solirubrobacter, Streptacidiphilus*	11
*Verrucomicrobia*	*Opitutus*	1	*Alterococcus, Opitutus*	2
*Chlamydiae*	*Neochlamydia, Parachlamydia, Rhabdochlamydia*	3	*Neochlamydia, Parachlamydia, Simkania*	3
*Chloroflexi*	*–*	0	*Ktedonobacter, Thermosporothrix*	2
*Elusimicrobia*	*–*	0	*Elusimicrobium*	1
*Firmicutes*	*Bacillus, Paenibacillus*	2	*Ammoniphilus, Bacillus, Brochothrix, Clostridium XI, Clostridium sensu stricto, Cohnella, Lactococcus, Lysinibacillus, Paenibacillus, Solibacillus, Sporosarcina, Viridibacillus*	12
*Gemmatimonadetes*	*Gemmatimonas*	1	*Gemmatimonas*	1
*Bacteroidetes*	*–*	0	*Flavobacterium, Mucilaginibacter, Niabella, Pedobacter, Sphingobacterium, Terrimonas*	6
*Nitrospira*	*Nitrospira*	1	*Nitrospira*	1
**Total genera**		**27**		**73**

Genera were identified at CT ≥0.8 using the online RDP classifier. *The genera *Byssovorax* and *Actinocorallia* were identified by OTU clustering.

Whereas OTU clustering resulted in 2% of the OTUs containing ∼50% of the total sequences (Turlapati et al., 2013), for oligotyping, 2% of the oligotypes contained ∼38% of the total number of sequences (results not shown). Another major difference was that 80% of the OTUs contained 10% of sequences in comparison to 20% sequences in 80% of oligotypes. A comparison of two databases (Greengenes used in QIIME vs. RDP online database) for classifying OTUs as well as oligotypes representative sequences resulted in a significantly lower number of taxa identified from both OTU and oligotype datasets with Greengenes as compared to RDP (**Table 4**). More genera were discernible when CT ≥0.5 was used (vs. ≥0.8) with oligotype representative sequences, (Table S4). One such example is the genus *Terriglobus* (*Acidobacteria-Gp*1), which was identified only at CT ≥0.5 (Figure S2).

**Table 4:** The effect of different databases on classification.

Database and tools comparisons for classification of oligo representatives (ORs) and operating taxonomic units OTUs				
**Method**	**Oligotyping**		**OTU clustering**	
**CT**	**0.8**		**0.8**	
**Tool**	**RDP classifier online**	**RDP in QIIME**	**RDP classifier online**	**RDP in QIIME**
**Database**	**RDP**	**Greengenes**	**RDP**	**Greengenes**
Actinobacteria	11	6	13	6
Bacteroidetes	6	2	3	1
Firmicutes	12	15	14	13
α*-Proteo*	11	6	14	11
β*-Proteo*	8	3	6	2
δ*-Proteo*	0	1	2	1
γ*-Proteo*	11	8	11	7
**Total**	**59**	**41**	**63**	**41**

*Oligotyping representative as well as OTU representative sequences were classified using the online RDP classifier version 2.2 (RDP database) and RDP version 2.2 within QIIME version 1.8.0 (Greengenes database). The OTU clustering was performed using UCLUST in QIIME with the identical parameters (*s* and *M*) that were set up for oligotyping. All genera identified at ≥0.8 CT*.

### BACTERIAL DIVERSITY ANALYSES

Details on the various steps of oligotyping analysis and the number of oligotypes identified for each phylum are given in Table S2. In general, no direct correlation was observed between the numbers of sequences and the oligotypes. In all, sequences affiliated with 73 known genera were identified from these soils as compared to 27 genera observed in our previous study (**Table 3**). Oligotyping revealed that sequences corresponding to some genera were present in control but absent in N-treated soils. In other instances, those present in N-amended plots were not found in control plots.

### BACTERIAL COMMUNITIES AND TREATMENT RELATIONSHIPS

**FIGURE 1:**
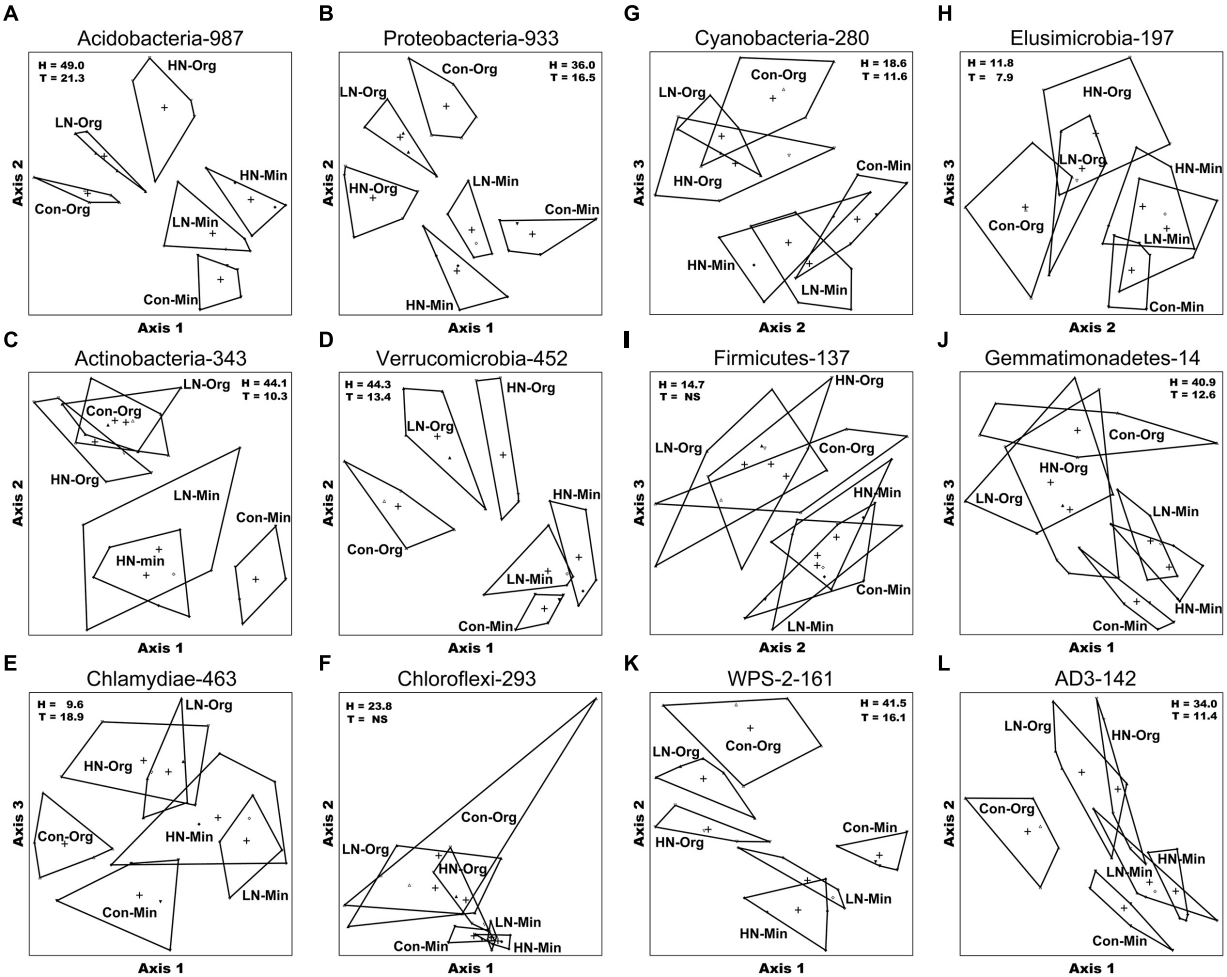
**Non-metric dimensional scaling (NMS) ordination for oligotypes of 30 soil samples for 12 of the 16 identified bacterial phyla.** Letter symbols refer to the NMS analyses for the following phyla: **(A)** Acidobacteria; **(B)** Proteobacteria; **(C)** Actinobacteria; **(D)** Verrucomicrobia; **(E)** Chlamydiae; **(F)** Chloroflexi; **(G)** Cyanobacteria; **(H)** Elusimicrobia; **(I)** Firmicutes; **(J)** Gemmatimonadetes; **(K)** WPS-2; and **(L)** AD3. Each soil type is represented by 5 replicates and a centroid, which is indicated by a single symbol and treatment-soil horizon name. The number next to the phylum name represents the total number of oligotypes identified within each phylum. H = % of variation partitioned by horizon and T = % of variation partitioned by treatment.

Non-metric multidimensional scaling (NMS) followed by permutational MANOVA (Permanova) with ordination scores, i.e., the Bray-Curtis distance, obtained from normalized total oligotype data revealed significant differences among bacterial communities based on treatment and soil horizon (**Figure 1**; Table S5). In general, all five replicate soil samples from within the same treatment plot clustered together and displayed stronger similarities among their oligotypes as compared to those from other treatment plots. For the two largest phyla, *Acidobacteria* and *Proteobacteria*, NMS and Permanova analyses were conducted for individual subgroups and classes, respectively (Figure S3; Table S5). With the exception of *Bacteroidetes* and subgroup *Gp*10 in *Acidobacteria,* the bacterial communities of the two horizons were significantly different. Additionally, within each soil horizon significant treatment effects on the structure of the bacterial community were observed for all phyla except for *Chloroflexi*, *Firmicutes*, TM7, *Bacteroidetes*, and subgroups *Gp*6 and *Gp*10 of *Acidobacteria* (**Figure 1** and Table S5; Figure S3).

The highest variation in partitioning was observed in the phylum *Acidobacteria,* where horizon explained 49.0% (*P* ≤ 0.0002) of the variation among samples, and treatment accounted for about 21% (*P* ≤ 0.0004) of the variation (**Figure 1A**; Table S5). Most of this variation was due to subgroups *Gp*1, *Gp*2, and *Gp*3 (Figure S3). In the second most diverse phylum, *Proteobacteria,* horizon explained 36% (*P* ≤ 0.0002) of variation among the samples and the treatment accounted for 16.5% (*P* ≤ 0.0006) of the variation; a major part of this variation was seen in *α*-and *γ-Proteobacteria* (Figure S3). Often there was an overlap between the LN- and HN-amended soil communities. Treatment effects were generally more pronounced in the Org soil horizon.

### SOIL CHARACTERISTICS AND BACTERIAL COMMUNITIES

Mantel test results on soil chemistry and the bacterial community revealed strong correlations for the Org soil. Pooled oligotyping data from all phyla found in the Org soil horizon showed a stronger positive correlation between the entire bacterial community and soil pH, Ca, P, K, Zn, Mg, NH_4_, NO_3_, and total C (**Table 5**) as compared to Min soil. When data for each phylum were analyzed separately, significant correlations were observed for *Acidobacteria*, *Proteobacteria*, *Actinobacteria*, *Verrucomicrobia,* WPS-2, and AD3. Exceptions included *Proteobacteria* which showed no correlation with Ca and P, nor did *Chlamydiae* with NH_4_ and total C. The remaining phyla had correlations with fewer elements, specifics of which varied (**Table 5**).

Oligotyping data pooled for all analyzed phyla from the Min horizon community showed strong positive correlations only with soil pH, acidity, NH_4_, and NO_3_. With few exceptions, this was true for analyses of each individual phylum. There was a positive correlation of Al with *Acidobacteria* and WPS-2, and Ca with *Proteobacteria* (**Table 5**).

**Table 5:** Relationship between soil chemistry and Bray-Curtis (Sorenson) distance measures of the normalized oligotypes data (Mantel test) conducted using PC-ORD software (version 6).

Soil Chemistry	pH	Ca	P	Mn	K	Zn	Mg	Acidity	Al	NH_4_	NO_3_	Total C
**Organic soil horizon**
All Bacteria	0.001*	0.007*	0.007*	-	0.004*	**0.001**	0.001*	-	-	0.015*	0.002*	0.002*
*Acidobacteria*	0.011*	0.028*	0.026*	**-**	0.012*	0.003*	0.002*	**-**	**-**	0.021*	0.001*	0.010*
*Proteobacteria*	0.027*	**-**	**-**	**-**	0.014*	0.001*	0.004*	**-**	**-**	0.041*	0.001*	0.037*
*Actinobacteria*	0.001*	0.006*	0.024*	**-**	**-**	0.021*	**-**	**-**	**-**	0.008*	0.024*	0.002*
*Verrucomicrobia*	0.001*	0.002*	0.012*	**-**	0.047*	0.004*	0.014*	**-**	**-**	0.003*	0.002*	0.006*
*Chlamydiae*	**-**	**-**	**-**	**-**	0.009*	0.011*	**-**	**-**	**-**	**-**	0.001*	**-**
*Chloroflexi*	**-**	**-**	0.001*	0.019*	0.015*	0.001*	0.006*	0.033*	**-**	0.013*	**-**	0.006*
*Cyanobacteria*	**-**	**-**	**-**	**-**	0.010*	0.009*	0.014*	**-**	**-**	**-**	0.001*	0.049*
*Elusimicrobia*	**-**	0.029*	**-**	**-**	**-**	**-**	**-**	**-**	**-**	0.011*	0.001*	-
*Firmicutes*	-	-	-	-	0.026*	0.017*	-	0.041*	-	0.021*	-	0.014*
*Gemmatimonadetes*	0.016*	0.008*	-	-	-	-	-	-	-	-	-	-
TM7	-	-	-	-	-	-	-	-	-	-	-	-
WPS-2	0.001*	0.023*	0.013*	-	0.003*	0.001*	0.010*	-	-	0.005*	0.002*	0.002*
AD3	0.008*	0.037*	0.002*	-	0.001*	0.001*	0.001*	0.035*	-	0.014*	0.001*	0.003*
*Bacteroidetes*	0.033*	-	0.017*	-	0.043*	-	-	0.016*	-	-	-	0.010*
**Mineral soil horizon**
* All Bacteria*	0.047*	-	-	-	-	-	-	0.020*	-	0.017*	0.005*	-
*Acidobacteria*	0.005*	-	-	-	-	-	-	0.009*	0.035*	0.037*	0.008*	-
*Proteobacteria*	-	0.009*	-	-	-	-	-	-	-	0.013*	0.005*	-
*Actinobacteria*	-	-	-	-	-	-	-	-	-	0.013*	-	-
*Verrucomicrobia*	0.029*	-	-	-	-	-	-	0.013*	-	0.031*	0.032*	-
*Chlamydiae*	-	-	-	-	-	0.040*	-	-	-	0.032*	0.001*	-
*Chloroflexi*	-	-	-	-	-	-	-	-	-	-	0.011*	-
*Cyanobacteria*	-	-	-	-	-	0.023*	-	-	-	-	0.002*	-
*Elusimicrobia*	-	0.041*	-	-	-	0.031*	-	-	-	0.050*	0.044*	-
*Firmicutes*	-	-	0.014*	-	-	0.024*	-	-	-	-	-	-
*Gemmatimonadetes*	0.016*	-	-	-	-	-	0.035*	0.041*	-	-	-	-
TM7	-	-	-	-	-	-	-	-	-	-	-	-
WPS-2	0.003*	-	-	-	-	-	0.026*	0.005*	0.009*	0.007*	0.010*	-
AD3	-	-	-	-	-	-	0.023*	-	-	-	-	-
*Bacteroidetes*	NA	NA	NA	NA	NA	NA	NA	NA	NA	NA	NA	NA

*All 15 samples from each soil horizon were pooled for these analyses. The iterations were set to 5000. Asterisks Indicates significant correlations (*P* ≤ 0.05). -Indicates non-significance. NA denotes that analyses could not be performed due to insufficient data*.

### CHANGES IN COMMUNITY STRUCTURE OF GENERA ASSOCIATED WITH SOIL HORIZON AND N-AMENDMENTS

**FIGURE 2:**
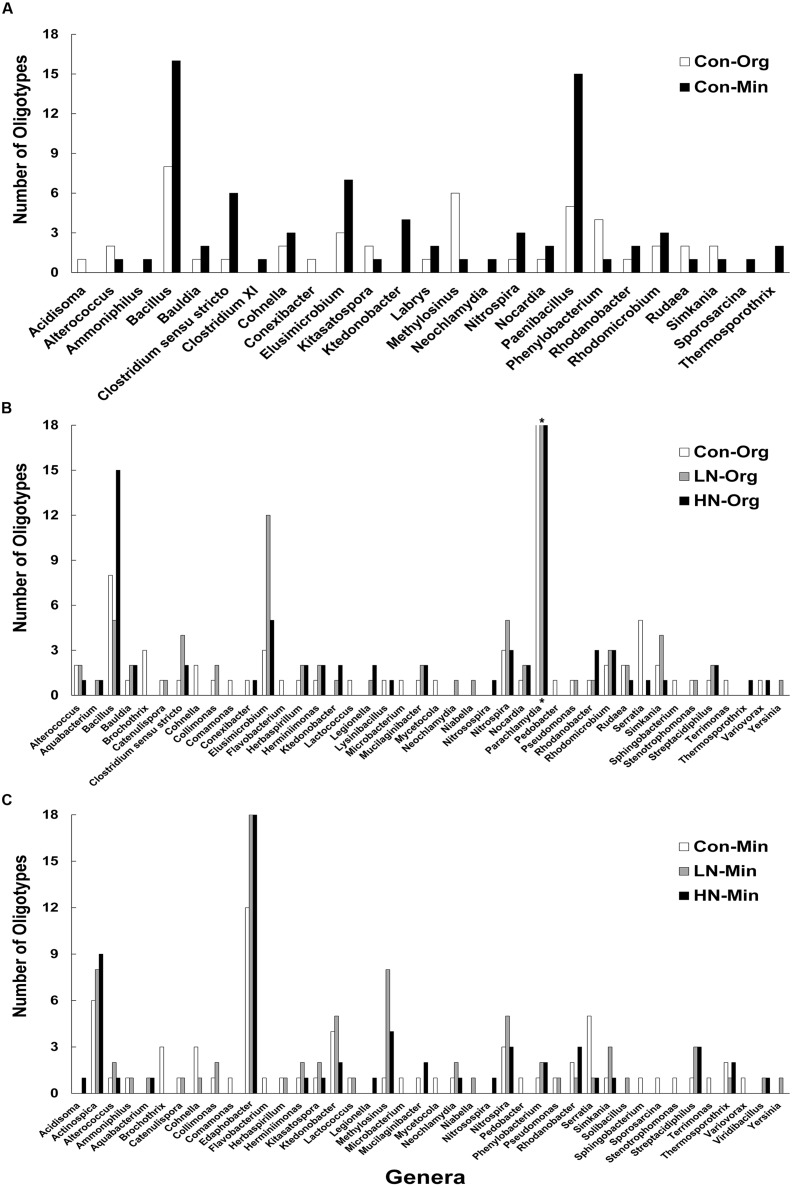
**Comparison of numbers of oligotypes for the identified genera in soil samples: **(A)** control organic vs. mineral soil; **(B)** control vs. N-amended organic soil; and **(C)** control vs. N-amended mineral soil.** The genera presented here were selected based on ≥50% change relative to control. Asterisk in **(B)** denotes the presence of >18 oligotypes for con (24), LN (44), and HN (39) for Parachlamydia.

Across all three treatments, oligotypes corresponding to five genera (*Ammoniphilus, Clostridium X1, Solibacillus, Sporosarcina,* and *Viridibacillus*) were found in Min soils but were absent in Org soils, and one genus (*Conexibacter*) was identified in Org soils but not in Min soils (**Table 6**; **Figures 2A,C**). Overall, oligotypes corresponding to five genera (*Aquabacterium, Nitrosospira, Yersinia, Legionella,* and *Niabella*) appeared exclusively in N-treated soils (in both horizons combined), while those representing eight genera (*Comamonas, Microbacterium, Mycetocola, Brochothrix, Flavobacterium, Pedobacter, Sphingobacterium,* and *Terrimonas*) were present in the control but absent from the N-treated soils (**Table 6**; **Figures 2B,C**). A comparison of the presence of oligotypes specific to each genus revealed that some oligotypes were unique to Org soils while others were exclusively present in Min soils (Table S6; **Figure 2**). Additionally some oligotypes were present only in the N-amended soils and not in the control plots. Although Figures and Tables show all identified genera, only those that exhibited significant differences with treatment or soil horizon are discussed in this section.

### TAXONOMY BY PHYLUM

*Acidobacteria,* which was the most abundant phylum in the HF soils, constituted ∼50% of total sequences and 22% of total oligotypes (Table S2). Subgroups *Gp*1, *Gp*2, and *Gp*3 accounted for most of the *Acidobacteria* sequences and oligotypes. Soil samples from control plots had three times more sequences affiliated with *Gp*1 as compared to *Gp*3. However, based on the total number of oligotypes, the *Gp*3 subgroup was more diverse than *Gp*1 in both soil horizons (Figures S4A,B). *Gp*13 was represented by a large number of oligotypes each containing small sets of sequences. Four new genera were identified in the phylum *Acidobacteria* since our last report with the same soil samples (**Table 3**). All of the genera identified in the Org horizon were represented by a significantly larger number of sequences as compared to those identified in the Min soil horizon for all three treatments (Figure S5A). However, the number of oligotypes did not vary greatly between soil horizons. Sequences of the genus *Edaphobacter* were significantly higher in HN vs. the control while *Acidobacterium*, *Granulicella*, and *Bryobacter* showed a reverse trend in the Org horizon (Figure S5A). Oligotypes for *Edaphobacter* were more abundant in N treatment plots relative to control in Min soil of both (**Figure 2C**).

*Proteobacteria* was the second most abundant phylum and was highly diverse in these soils. This phylum comprised ∼20% of the total sequences and total oligotypes (Table S2). Classes α-*Proteobacteria* and γ-*Proteobacteria* together accounted for >70% of this phylum’s sequences and oligotypes in each horizon (Figures S6A,B). Regardless of the number of sequences, oligotyping data revealed that among all other classes and phyla, α-*Proteobacteria* were the most diverse in both soil horizons. In general, LN treatment was positively correlated with sequences and oligotype numbers. Altogether, 30 genera were found in *Proteobacteria* (Figures S5B–D, **Table 3**): 11 each in α*-* and γ-*Proteobacteria* (Figures S5B–D), and eight in β*-Proteobacteria* (Figure S5C). Fourteen out of these 30 genera were present in relatively high abundance (sequences ≥50). Shifts in community structure were observed among most genera in response to N treatments (Figures S6A,B). An increase in the number of sequences was observed for several genera in soil treated with LN (Figures S5B,D).

*Actinobacteria* constituted only 7% of total sequences and 7.6% of oligotypes (Table S2). Eleven genera were identified from this phylum relative to the prior findings of seven (by OTU clustering) for the same data set (**Table 3**). The absence of certain genera in N-amended soils was observed in a few cases (Figure S5E).

*Verrucomicrobia* was the third most abundant phylum with ∼10% of sequences and ∼10% of oligotypes in these soils (Table S2). Only two genera (**Table 3**) from this phylum were identified. Genus *Opitutus* was present in tenfold higher numbers than *Alterococcus* (Figure S5F). The number of sequences for *Opitutus* was lower in N-treated soils than in Control. For both *Actinobacteria* and *Verrucomicrobia*, horizon and treatment explained 44 and 10–13% of the total variation, respectively (**Figures 1C,D**).

*Chlamydiae* constituted 2.5% of the total sequences and ∼10% of oligotypes (Table S2). Three genera were identified in this phylum (**Table 3**); the relative abundance of oligotypes corresponding to the genus *Parachlamydia* were highest in N treatment plots relative to control in Org soil (**Figure 2B**; Figure S5G).

*Chloroflexi* constituted 2.7% of the total sequences and 6.5 % of oligotypes (Table S2), with only two genera (*Ktedonobacter* and *Thermosporothrix* – Figure S5H; **Table 3**), which were absent in the control treatment in the Org horizon but present in the Min soils (**Figure 2A**).

Although *Firmicutes* constituted only about 0.3% of total sequences, with 3.0% oligotypes, 12 genera were identified in this phylum (Table S2; Figure S5J). Most of these were present in very low numbers and were more prevalent in Min soils (e.g., *Bacillus* and *Paenibacillus*); all five of the genera seen in the Min soil horizon were absent in the Org soil horizon (**Table 6**; **Figure 2**); there was little effect of N treatments (**Figure 1I**; **Table 5**).

**Table 6:** Horizon specific genera (shown with an *) and genera that appeared or disappeared with N-amendments.

Phylum or class	Con-Org	LN-Org	HN-Org	Con-Min	LN-Min	HN-Min
*Actinobacteria*	Conexibacter* Microbacterium *Mycetocola*	-	Conexibacter*	*Microbacterium* *Mycetocola*	-	-
*Firmicutes*	*Brochothrix*	-	-	Ammoniphilus* Brochothrix Clostridium XI* Sporosarcina*	Ammoniphilus* Clostridium XI* Solibacillus* Viridibacillus*	Clostridium XI* Viridibacillus*
β*-Proteobacteria*	*Comamonas*	*Aquabacterium*	*Aquabacterium Nitrosospira*	*Comamonas*	*Aquabacterium*	*Aquabacterium* *Nitrosospira*
γ*-Proteobacteria*	-	*Legionella, Yersinia*	*Legionella*	-	*Yersinia*	*Legionella*
*Bacteroidetes*	*Flavobacterium* *Pedobacter* *Sphingobacterium* *Terrimonas*	*Niabella*	-	*Flavobacterium* *Pedobacter* *Sphingobacterium* *Terrimonas*	*Niabella*	-

Together the phyla TM7, *Gemmatimonadetes,* and *Nitrospira* comprised ∼1% of the total sequences and 2.4% of oligotypes (Table S2). From these three phyla, only two genera were identified; *Gemmatimonas* in *Gemmatimonadetes* and *Nitrospira* in *Nitrospira* (Figures S5L,M). The number of sequences representing the genus *Nitrospira* was greater in Min vs. the Org soil horizon and in LN treatment vs. the control and HN treatment. While both treatment and horizon explained significant variation found in *Gemmatimonadetes* (**Figure 1J**; Table S5) only horizon-specific effects were seen for TM7 (Figure S3L). NMS generated a four dimensional solution for TM7 (Table S5). Because of a small dataset, no further analyses were conducted on *Nitrospira*.

The phyla TM6*, Cyanobacteria, Elusimicrobia,* WPS-2*,* and AD3 were not identified in our previous report with the same data. TM6 was represented by only 36 sequences, most of which were filtered out in the initial oligotyping run and, therefore, were not analyzed further (Table S2). The other five phyla together constituted a relatively small portion of the total sequences (∼5.2%, with numbers ranging from 142 to 280) and 17.2% of the total oligotypes (Table S2). Among these phyla, WPS-2 was the most affected by treatment as well as by horizon (**Figure 1K**). Only the genus *Elusimicrobium* was identified in *Elusimicrobia* (Figure S5I).

*Bacteroidetes* contained only 259 sequences but 36 oligotypes, and six distinct genera were identified in this phylum; four of which were absent from N-amended soils (**Table 6**; Figure S5K). NMS analyses yielded only one dimension solution for this phylum, and thus no figure was generated (Table S5).

## DISCUSSION

The primary objective for re-analyses of the pyrosequencing data from the previous study (Turlapati et al., 2013) was to determine additional diversity by applying more efficient and reliable bioinformatics tools. The results enabled us to identify a total of 4534 oligotypes belonging to 15 different bacterial phyla with 73 genera. The same dataset had previously resulted in 6936 OTUs belonging to 11 different bacterial phyla with only 27 identifiable genera. There are two main explanations for this apparent discrepancy: the first being the updating of tools and databases used for classification to newer versions since our previous report; and the second (somewhat obscure) is the difference between the classifiers and databases currently being used for the two clustering methods. Whereas the RDP classifier version 2.2 in QIIME pipeline uses the Greengenes database for OTU- rep classification, the RDP online classifier version 2.7 used for oligotyping has its own built-in RDP database. Although oligotyping and OTU clustering identify similar numbers of taxa (in a comparative study using four phyla constituting ∼28% of total sequences, **Table 4**), the former has greater resolving ability for classifying nearly identical, closely related organisms provided a good reference genomic library is available to compare against. Using entropy analysis, oligotyping simultaneously clusters multiple sequences based on similarity/identity of each nucleotide along the entire length of the read for all sequences within a given group. However, OTU groups sequences at 97% similarity with a representative sequence. The 3% difference in nucleotides may occur anywhere along the entire length of the read, and that location can vary from sequence to sequence within the group. That is to say, 2 sequences within an OTU can differ from the representative sequence at different nucleotide positions. Additionally, once a sequence has been selected and grouped within one OTU it cannot be assigned to another even if it has greater similarity with the representative sequence of the second OTU. It is this difference in the way sequences are clustered by these methods that makes oligotyping more powerful in grouping closely related organisms. While a relationship between oligotypes and most soil chemistry parameters was observed only a few such relationships were observed for OTU data by the earlier report (Turlapati et al., 2013).

A major limitation of any study involving a soil microbiome (including the present one) is the lack of reference genomes (even for dominant taxa; Howe et al., 2014). Metagenomic outputs of most current high-throughput sequencing technologies (e.g., Illumina) often result in a mixture of multiple genomes most of which do not cover a complete genome of the organisms of interest since complete reference genomes of known organisms are lacking (Simon and Daniel, 2011; Teeling and Glöckner, 2012). Therefore, many studies still rely on universally occurring DNA sequences (either partial or complete), e.g., the 16S rRNA genes. Single cell genomics offers a powerful technique for characterizing the genome of a single organism (Zaremba-Niedzwiedzka et al., 2013; Macaulay and Voet, 2014); however, this is still an emerging technology and is difficult to apply to the microbiome of complex systems like forest soils. In the absence of genome-specific sequence libraries from forest soils, it is difficult to assign the terminal taxonomic identity (e.g., at species level) even to 16S rRNA genes or any other gene sequences. Thus, amplicon-sequencing (although known to be biased against rare organisms) remains a realistic approach to estimate the diversity of large microbial populations in complex environments.

Oligotyping enables the detection and classification of distinct subpopulations within a genus, or even within a single species as was shown for *Gardnerella vaginalis* in humans (Eren et al., 2011). With forest soil microbiomes, although taxonomy at the species level could not be assigned because of the lack of reference genome data, oligotyping did enable us to detect subpopulations within a genus. Furthermore, the distribution of many of these subpopulations often varied between the soil horizons and among long-term treatments with N fertilizer (Table S6).

The results presented here demonstrate the applicability of oligotyping to complex microbiomes of forest soils. However, some adjustments may be necessary to the stringency of parameters that Eren et al. (2013) had suggested. For example, in order to assess the diversity of closely related bacterial populations in an ecosystem by oligotyping of 16S rRNA gene sequences, Eren et al. (2013) emphasized the importance of at least four critical parameters (namely ‘s,’ ‘a,’ ‘A’ and ‘M’) that minimize the impact of sequencing errors in determining the outcome of results. They further summarized that s and M are critical components used to reduce the noise in such analyses. Soil samples have greater microsite variability, bacterial diversity and the occurrence of rare organisms as compared to other microbiomes (e.g., human body and marine waters). All of the data in the present study were analyzed using *M* values based on two criteria: namely, retaining maximum sequences and the diversity in terms of the number of oligotypes with an *s* = 2 (due to high microsite variability among five replicates). In the present study, high diversity was evident from a large number of entropy peaks with high values for components for most phylum level datasets (Figure S1). Therefore, oligotyping of these soil samples required several rounds of supervised (user-defined component selection) analyses before all of the entropy peaks could be decomposed. Lowering the *M* values from those suggested by Eren et al. (2013), especially for relatively larger datasets led to the identification of more genera at CT ≥0.8 (**Table 1**). This suggests that many organisms are probably present in low abundance (constituting the rare microbiome) in HF soil. Even with much smaller datasets, where *M* values of two or three were used, genera were identified at CT ≥0.8 (e.g., genus *Aquabacterium* of class β*-Proteobacteria* – **Table 1**). Huse et al. (2010) suggested that if a sequence occurs in two separate environmental samples (i.e., *s* = 2), then the chance of it being noise or a technical error is almost zero and thus should be considered as a sequence affiliated with a rare organism. Using this same dataset, Turlapati et al. (2013) earlier reported 4093 singleton OTUs among a total of 11,029 (37%) with *s* = 1 (default). Therefore, the present classification with oligotyping with *s* = 2 should be more reliable as compared to OTU clustering in eliminating noise.

The primers used in the present study specifically target the V6–V8 region of 16S rRNA genes and were chosen due to the high sequence variability associated with this region (Brons and van Elsas, 2008). The poor ability of RDP to assign taxonomy to V6 reads at CT ≥0.8 as compared to CT ≥0.5 has been reported by Claesson et al. (2009). Similarly, in this present study, a greater number of genera were identified at CT ≥0.5 vs. CT ≥0.8 (Table S5). For example, genus *Terriglobus* (*Acidobacteria, Gp1*) could only be identified at CT ≥0.5; sequences for this genus were found in all 30 samples and were significantly higher in HN-Org soils as compared to control. These observations suggest that the standard CT value of ≥0.8 at the genus level may need to be adjusted when working with the V6-V8 hypervariable regions of the 16S rRNA gene especially in ecosystems for which reference genome libraries are lacking.

Available analytical tools and public databases, such as RDP, are constantly being updated to meet increasing demand for taxonomic classification arising from high throughput outputs created by next generation sequencing platforms (Cole et al., 2009, 2013). Mclean et al. (2013) reported 31 candidate phyla including recently identified TM6 in the bacterial population of a hospital sink. In the present study, A total of 16 phyla were identified as compared to 11 in our previous report which used the same dataset in the QIIME pipeline (Turlapati et al., 2013). The RDP classifier assigned phyla names such as AD3, *Elusimicrobia, Cyanobacteria*, TM6, WPS-2 to the sequences that were termed unclassified in our previous study. No genera were identified within these phyla with the exception of *Elusimicrobium*. Most importantly, the overall unclassified sequences previously constituting 15–20% of the total were reduced to 0.5% in the current analysis.

Although *Acidobacteria* constituted >50% of total sequences, only four genera were identified in this phylum. The availability of reference genomes would be useful in further classifying this phylum; however, to date only eight genera have been taxonomically described in this phylum (Männistö et al., 2011 and references therein). Naether et al. (2012) reported that within *Acidobacteria Gp*1, *Gp*2, and *Gp*3 organisms favor nutrient-limited soils as compared to other subgroups. The dominance of these three subgroups of *Acidobacteria* in both soil horizons at HF suggests that these soils are perhaps nutrient limited. VanInsberghe et al. (2013) reported that in comparison with *Proteobacteria, Acidobacteria* are more prevalent in soils with low resource availability; our results are in agreement with this report and further reinforce the conclusion that HF soils are nutrient poor. Differences observed between bacterial communities in the Org and Min soil are clearly attributable to the differences in the soil chemistry of the two horizons (Table S3). Although HF soils may be nutrient limited, bacteria in the Org horizon are perhaps adapted to relatively nutrient-rich environment compared to those in the Min horizon. Our results demonstrate that with few exceptions, the Org soil communities were more impacted by N-treatment as compared to the Min soil communities. Compared to Min soil horizon, bacteria in the Org soils demonstrated stronger relationships with most of the soil chemistry parameters (**Table 5**). Fierer et al. (2012) also reported greater phylogenetic shifts in microbial communities that prefer a nutrient rich environment following N fertilization.

Naether et al. (2012) also reported correlations between edaphic factors such as pH, C, N, C/N ratio, and P with corresponding OTUs (16S clones) and terminal-RFLP (T-RFLP) for most of the subgroups of *Acidobacteria* found in soil from 30 forested and 27 grassland sites. They found either positive or negative correlations of different OTUs or T-RFLPs within respective subgroups of *Acidobacteria* over a wide pH and nutrient range. Another study involving 87 soil types with pH values ranging from 3.5 to 8.5 reported an overall inverse relationship between soil pH and the relative abundance of *Acidobacteria* (Jones et al., 2009). However, a closer look at data shows that within a narrow range of pH from 3.5 to 4.5, this inverse relationship is not held. The pH of HF soil ranged from 3.8 to 4.4 for Org and 4.3 to 4.8 for Min. At HF, a positive correlation between the subgroups of *Acidobacteria* and pH in this narrow range for each soil horizon indicates that for optimal growth, this group prefers the higher end of this narrow range at HF (Table S3). Our results are in agreement with those of Sait et al. (2006) who found that subgroup *Gp*1 ideally requires moderately acidic conditions (pH 4.0–5.5).

Despite the existence of significant effects on aboveground foliar Org N metabolites (polyamines and amino acids; Table S3), tree physiology and productivity (Minocha et al., 2000; Bauer et al., 2004; Magill et al., 2004; Frey et al., 2014), and changes in soil microbial diversity at the HF, the lack of any lasting effects of N-amendment on soil NH_4_ and NO_3_ concentrations is interesting and apparently contradictory. We speculate that this is due to the combined effects of the fast uptake of the fertilizer (applied only during the growing season) by the macroflora, its rapid conversion into other inorganic N and Org N metabolites (e.g., polyamines and amino acids), and leaching of a significant amount of applied N.

Polyamines are present in all living organisms. They are required for growth and are also involved in stress responses (Minocha et al., 2014). Polyamines and specific-amino acids (e.g., glutamine and arginine) are known to be major N storage metabolites in plants, especially under excess N conditions. Concentrations of these compounds were found to be high in the foliage of trees growing in the N-treated plots at the HF (Minocha et al., 2000; Bauer et al., 2004). Changes in concentrations of polyamines and amino acids were observed in the same soils used for the present study (Frey et al., 2014). These findings suggest that the effects of N-addition on shifts in bacterial community structure may have resulted partially through effects of N-amendments on the growth of the aboveground plant community and vice versa. Also, it can be hypothesized that changes observed in the microbiome are due to the preference of certain microbial taxa for excess N that was present immediately following N application. Then, over the longer term of repeated N applications, they were stabilized and became a major component of the microbiome during the phase when soil inorganic N reverted back to original levels. This argument is supported by the observation that some of the functionally important genera (e.g., *Nitrosospira* of *phylum Proteobacteria*, which include well-known NH_3_ oxidizers/nitrifiers) appeared mostly in N-treated soils. Using the amplification of a functional N-transformation gene *amo*A, He et al. (2007) observed an increased abundance of *Nitrosospira* sequences in response to N-treatments at a Chinese Agricultural Experimental Station. In our study the sequences and oligotypes corresponding to *Nitrospira* were higher in LN-treated Min soil. However, using 16S markers, Wertz et al. (2012) observed no change in the abundance of sequences for *Nitrospira* (another potential nitrifier group within the phylum *Nitrospira*) in response to long term N-fertilization at five forested sites in British Columbia, Canada.

## CONCLUSION

A total of 46 previously unidentified genera were recognized by oligotyping vs. OTU clustering analysis of PCR-amplified partial 16S rDNA sequences from HF soil DNA. Because of the lack of a reference genome database for forest soils, both clustering approaches yield limited information at the genus and species level; however, oligotyping enables reliable classification of closely related organisms because of the high stringency of this tool. This analytical approach further revealed strong correlations between soil chemistry and oligotypes; no such correlations were discernible with the OTU clustering approach. Based on the fact that we could identify several genera at CT ≥0.98 using a relatively lower *M* value, we suggest that lowering *M* values may be appropriate for the complex microbiomes such as forest soils that are comprised of an enormous diversity of bacteria that are often present in low abundance. As suggested by Mantel test results, bacterial communities in the Org soil at HF have high preference for a nutrient rich environment and the communities found in the Min soil are better adapted to nutrient poor conditions. Overall, effects of N-treatment on the microbiome were more evident in the Org soil than the Min soil horizon, perhaps due to the fact that N utilization requires an abundance of C, which three times higher in the Org as compared to Min soil.

## Conflict of Interest Statement

The authors declare that the research was conducted in the absence of any commercial or financial relationships that could be construed as a potential conflict of interest.

## Footnotes

1 http://harvardforest.fas.harvard.edu/research/LTER

2 http://harvardforest.fas.harvard.edu/

3 http://anlab.umesci.maine.edu

4 http://www.biotech.illinois.edu/htdna

5 http://sourceforge.net/projects/rdp-classifier/

6 http://sourceforge.net/projects/rdp-classifier/files/rdp-classifier/rdp_classifier_2.7.zip/download

7 http://www.ebi.ac.uk/Tools/msa/clustalw2/

## References

[B1] Aber J. D. Alison M. Boone R. Melillo J. M. Steudler P. (1993). Plant and soil responses to chronic nitrogen additions at the Harvard Forest, Massachusetts. *Ecol. Appl.* 3 156–166 10.2307/1941798 27759220

[B2] Bauer G. A. Bazzaz F. A. Minocha R. Long S. Magill A. Aber J. (2004). Effects of chronic N additions on tissue chemistry, photosynthetic capacity, and carbon sequestration potential of a red pine (*Pinus resinosa Ait.*) stand in the NE United States. *For. Ecol. Manage.* 196 173–186 10.1016/j.foreco.2004.03.032

[B3] Bischoff J. Mangelsdorf K. Schwamborn G. Wagner D. (2014). Impact of lake-level and climate changes on microbial communities in a terrestrial permafrost sequence of the El’gygytgyn Crater, far East Russian Arctic. *Permafrost Periglacial Process.* 25 107–116 10.1002/ppp.1807

[B4] Bowden R. D. Davidson E. Savage K. Arabia C. Steudler P. (2004). Chronic nitrogen additions reduce total soil respiration and microbial respiration in temperate forest soils at the Harvard Forest. *For. Ecol. Manage.* 196 43–56 10.1016/j.foreco.2004.03.011

[B5] Brons J. K. van Elsas J. D. (2008). Analysis of bacterial communities in soil by use of denaturing gradient gel electrophoresis and clone libraries, as influenced by different reverse primers. *Appl. Environ. Microbiol.* 74 2717–2727 10.1128/AEM.02195-07 18310425 PMC2394888

[B6] Caporaso J. G. Bittinger K. Bushman F. D. Desantis T. Z. Andersen G. L. Knight R. (2010a). PyNAST: a flexible tool for aligning sequences to a template alignment. *Bioinformatics* 26 266–267 10.1093/bioinformatics/btp636 19914921 PMC2804299

[B7] Caporaso J. G. Kuczynski J. Stombaugh J. Bittinger K. Bushman F. D. Costello E. K. (2010b). QIIME allows analysis of high-throughput community sequencing data. *Nat. Methods* 7 335–336 10.1038/nmeth.f.303 20383131 PMC3156573

[B8] Carney K. M. Matson P. A. (2006). The influence of tropical plant diversity and composition on soil microbial communities. *Microb. Ecol.* 52 226–238 10.1007/s00248-006-9115-z 16897297

[B9] Claesson M. J. O’sullivan O. Wang Q. Nikkilä J. Marchesi J. R. Smidt H. (2009). Comparative analysis of pyrosequencing and a phylogenetic microarray for exploring microbial community structures in the human distal intestine. *PLoS ONE* 4:e6669 10.1371/journal.pone.0006669 PMC2725325 19693277

[B10] Cole J. R. Wang Q. Cardenas E. Fish J. Chai B. Farris R. J. (2009). The ribosomal database project: improved alignments and new tools for rRNA analysis. *Nucleic Acids Res.* 37 D141–D145 10.1093/nar/gkn879 19004872 PMC2686447

[B11] Cole J. R. Wang Q. Fish J. A. Chai B. Mcgarrell D. M. Sun Y. (2013). Ribosomal Database Project: data and tools for high throughput rRNA analysis. *Nucleic Acids Res.* 42 D633–D642 10.1093/nar/gkt1244 24288368 PMC3965039

[B12] Compton J. E. Watrud L. S. Arlene Porteous L. Degrood S. (2004). Response of soil microbial biomass and community composition to chronic nitrogen additions at Harvard forest. *For. Ecol. Manage.* 196 143–158 10.1016/j.foreco.2004.03.017

[B13] Coolon J. D. Jones K. L. Todd T. C. Blair J. M. Herman M. A. (2013). Long-term nitrogen amendment alters the diversity and assemblage of soil bacterial communities in Tallgrass Prairie. *PLoS ONE* 8:e67884 10.1371/journal.pone.0067884 PMC3695917 23840782

[B14] Currie W. S. Aber J. D. Driscoll C. T. (1999). Leaching of nutrient cations from the forest floor: effects of nitrogen saturation in two long-term manipulations. *Can. J. For. Res.* 29 609–620 10.1139/x99-033

[B15] Cusack D. F. Silver W. L. Torn M. S. Burton S. D. Firestone M. K. (2010). Changes in microbial community characteristics and soil organic matter with nitrogen additions in two tropical forests. *Ecology* 92 621–632 10.1890/10-0459.1 21608471

[B16] Edgar R. C. (2010). Search and clustering orders of magnitude faster than BLAST. *Bioinformatics* 26 2460–2461 10.1093/bioinformatics/btq461 20709691

[B17] Eren A. M. Maignien L. Sul W. J. Murphy L. G. Grim S. L. Morrison H. G. (2013). Oligotyping: differentiating between closely related microbial taxa using 16S rRNA gene data. *Methods Ecol. Evol.* 4 1111–1119 10.1111/2041-210X.12114 PMC3864673 24358444

[B18] Eren A. M. Zozaya M. Taylor C. M. Dowd S. E. Martin D. H. Ferris M. J. (2011). Exploring the diversity of *Gardnerella vaginalis* in the genitourinary tract microbiota of monogamous couples through subtle nucleotide variation. *PLoS ONE* 6:e26732 10.1371/journal.pone.0026732 PMC3201972 22046340

[B19] Falkowski P. G. Fenchel T. Delong E. F. (2008). The microbial engines that drive earth’s biogeochemical cycles. *Science* 320 1034–1039 10.1126/science.1153213 18497287

[B20] Fierer N. Jackson R. B. (2006). The diversity and biogeography of soil bacterial communities. *Proc. Natl. Acad. Sci. U.S.A.* 103 626–631 10.1073/pnas.0507535103 16407148 PMC1334650

[B21] Fierer N. Lauber C. L. Ramirez K. S. Zaneveld J. Bradford M. A. Knight R. (2012). Comparative metagenomic, phylogenetic and physiological analyses of soil microbial communities across nitrogen gradients. *ISME J.* 6 1007–1017 10.1038/ismej.2011.159 22134642 PMC3329107

[B22] Frey S. D. Knorr M. Parrent J. L. Simpson R. T. (2004). Chronic nitrogen enrichment affects the structure and function of the soil microbial community in temperate hardwood and pine forests. *For. Ecol. Manage.* 196 159–171 10.1016/j.foreco.2004.03.018

[B23] Frey S. D. Ollinger S. Nadelhoffer K. Bowden R. Brzostek E. Burton A. (2014). Chronic nitrogen additions suppress decomposition and sequester soil carbon in temperate forests. *Biogeochemistry* 121 305–316 10.1007/s10533-10014-10004-10530

[B24] Fulthorpe R. R. Roesch L. F. W. Riva A. Triplett E. W. (2008). Distantly sampled soils carry few species in common. *ISME J.* 2 901–910 10.1038/ismej.2008.55 18528413

[B25] Gloor G. B. Hummelen R. Macklaim J. M. Dickson R. J. Fernandes A. D. Macphee R. (2010). Microbiome profiling by illumina sequencing of combinatorial sequence-tagged PCR products. *PLoS ONE* 5:e15406 10.1371/journal.pone.0015406 PMC2964327 21048977

[B26] Gruber N. Galloway J. N. (2008). An earth-system perspective of the global nitrogen cycle. *Nature* 451 293–296 10.1038/nature06592 18202647

[B27] Hallin S. Jones C. M. Schloter M. Philippot L. (2009). Relationship between N-cycling communities and ecosystem functioning in a 50-year-old fertilization experiment. *ISME J.* 3 597–605 10.1038/ismej.2008.128 19148144

[B28] He J.-Z. Shen J.-P. Zhang L.-M. Zhu Y.-G. Zheng Y.-M. Xu M.-G. (2007). Quantitative analyses of the abundance and composition of ammonia-oxidizing bacteria and ammonia-oxidizing archaea of a Chinese upland red soil under long-term fertilization practices. *Environ. Microbiol.* 9 2364–2374 10.1111/j.1462-2920.2007.01358.x 17686032

[B29] Howe A. C. Jansson J. K. Malfatti S. A. Tringe S. G. Tiedje J. M. Brown C. T. (2014). Tackling soil diversity with the assembly of large, complex metagenomes. *Proc. Natl. Acad. Sci. U.S.A.* 111 4904–4909 10.1073/pnas.1402564111 24632729 PMC3977251

[B30] Huse S. M. Dethlefsen L. Huber J. A. Welch D. M. Relman D. A. Sogin M. L. (2009). Exploring microbial diversity and taxonomy using SSU rRNA hypervariable tag sequencing. *PLoS Genet.* 4:e1000255 10.1371/journal.pgen.1000255 PMC2577301 19023400

[B31] Huse S. M. Welch D. M. Morrison H. G. Sogin M. L. (2010). Ironing out the wrinkles in the rare biosphere through improved OTU clustering. *Environ. Microbiol.* 12 1889–1898 10.1111/j.1462-2920.2010.02193.x 20236171 PMC2909393

[B32] Janssen P. H. (2006). Identifying the dominant soil bacterial taxa in libraries of 16S rRNA and 16S rRNA genes. *Appl. Environ. Microbiol.* 72 1719–1728 10.1128/AEM.72.3.1719-1728.2006 16517615 PMC1393246

[B33] Janssens I. A. Dieleman W. Luyssaert S. Subke J. A. Reichstein M. Ceulemans R. (2010). Reduction of forest soil respiration in response to nitrogen deposition. *Nat. Geosci.* 3 315–322 10.1038/ngeo844

[B34] Jones R. T. Robeson M. S. Lauber C. L. Hamady M. Knight R. Fierer N. (2009). A comprehensive survey of soil acidobacterial diversity using pyrosequencing and clone library analyses. *ISME J.* 3 442–453 10.1038/ismej.2008.127 19129864 PMC2997719

[B35] Langenfeld A. Prado S. Nay B. Cruaud C. Lacoste S. Bury E. (2013). Geographic locality greatly influences fungal endophyte communities in *Cephalotaxus harringtonia*. *Fungal Biol.* 117 124–136 10.1016/j.funbio.2012.12.005 23452950

[B36] Lauber C. L. Hamady M. Knight R. Fierer N. (2009). Pyrosequencing-based assessment of soil pH as a predictor of soil bacterial community structure at the continental scale. *Appl. Environ. Microbiol.* 75 5111–5120 10.1128/AEM.00335-09 19502440 PMC2725504

[B37] Lauber C. L. Strickland M. S. Bradford M. A. Fierer N. (2008). The influence of soil properties on the structure of bacterial and fungal communities across land-use types. *Soil Biol. Biochem.* 40 2407–2415 10.1016/j.soilbio.2008.05.021

[B38] Lombard N. Prestat E. Van Elsas J. D. Simonet P. (2011). Soil-specific limitations for access and analysis of soil microbial communities by metagenomics. *FEMS Microbiol. Ecol.* 78 31–49 10.1111/j.1574-6941.2011.01140.x 21631545

[B39] Long R. P. Horsley S. B. Lilja P. R. (1997). Impact of forest liming on growth and crown vigor of sugar maple and associated hardwoods. *Can. J. For. Res.* 27 1560–1573 10.1139/x97-074

[B40] Lynch M. D. J. Bartram A. K. Neufeld J. D. (2012). Targeted recovery of novel phylogenetic diversity from next-generation sequence data. *ISME J.* 6 2067–2077 10.1038/ismej.2012.50 22791239 PMC3475379

[B41] Macaulay I. C. Voet T. (2014). Single cell genomics: advances and future perspectives. *PLoS Genet.* 10:e1004126 10.1371/journal.pgen.1004126 PMC3907301 24497842

[B42] Magill A. H. Aber J. D. Currie W. S. Nadelhoffer K. J. Martin M. E. Mcdowell W. H. (2004). Ecosystem response to 15 years of chronic nitrogen additions at the Harvard Forest LTER, Massachusetts, USA. *For. Ecol. Manage.* 196 7–28 10.1016/j.foreco.2004.03.033

[B43] Männistö M. K. Rawat S. Starovoytov V. Häggblom M. M. (2011). Terriglobus saanensis sp. nov., an *acidobacterium* isolated from tundra soil. *Int. J. Syst. Evol. Microbiol.* 61 1823–1828 10.1099/ijs.0.026005-0 21186292

[B44] Margulies M. Egholm M. Altman W. E. Attiya S. Bader J. S. Bemben L. A. (2005). Genome sequencing in microfabricated high-density picolitre reactors. *Nature* 437 376–380 10.1038/nature03959 16056220 PMC1464427

[B45] McDonald D. Price M. N. Goodrich J. Nawrocki E. P. Desantis T. Z. Probst A. (2012). An improved greengenes taxonomy with explicit ranks for ecological and evolutionary analyses of bacteria and archaea. *ISME J.* 6 610–618 10.1038/ismej.2011.139 22134646 PMC3280142

[B46] McGuire K. Fierer N. Bateman C. Treseder K. Turner B. (2012). Fungal community composition in neotropical rain forests: the influence of tree diversity and precipitation. *Microb. Ecol.* 63 804–812 10.1007/s00248-011-9973-x 22080256

[B47] Mclean J. S. Lombardo M.-J. Badger J. H. Edlund A. Novotny M. Yee-Greenbaum J. (2013). Candidate phylum TM6 genome recovered from a hospital sink biofilm provides genomic insights into this uncultivated phylum. *Proc. Natl. Acad. Sci. U.S.A.* 110 E2390–E2399 10.1073/pnas.1219809110 23754396 PMC3696752

[B48] Minocha R. Long S. L. Magill A. Aber J. Mcdowell W. (2000). Foliar free polyamine and inorganic ion content in relation to soil and soil solution chemistry in two fertilized forest stands at the Harvard Forest, Massachusetts. *Plant Soil* 222 119–137 10.1023/A:1004775829678

[B49] Minocha R. Long S. Thangavel P. Minocha S. C. Eagar C. Driscoll C. T. (2010). Elevation dependent sensitivity of northern hardwoods to Ca addition at Hubbard Brook experimental forest, NH USA. *For. Ecol. Manage.* 260 2115–2125 10.1016/j.foreco.2010.09.002

[B50] Minocha R. Majumdar R. Minocha S. C. (2014). Polyamines and abiotic stress in plants: A complex relationship. *Front. Plant Sci.* 5:175 10.3389/fpls.2014.00175 PMC4017135 24847338

[B51] Naether A. Foesel B. U. Naegele V. Wust P. K. Weinert J. Bonkowski M. (2012). Environmental factors affect Acidobacterial communities below the subgroup level in grassland and forest soils. *Appl. Environ. Microbiol.* 78 7398–7406 10.1128/AEM.01325-12 22885760 PMC3457104

[B52] Nunes da Rocha U. Van Overbeek L. Van Elsas J. D. (2009). Exploration of hitherto-uncultured bacteria from the rhizosphere. *FEMS Microbiol. Ecol.* 69 313–328 10.1111/j.1574-6941.2009.00702.x 19508698

[B53] Ramirez K. S. Craine J. M. Fierer N. (2012). Consistent effects of nitrogen amendments on soil microbial communities and processes across biomes. *Glob. Chang. Biol.* 18 1918–1927 10.1111/j.1365-2486.2012.02639.x

[B54] Ramirez K. S. Lauber C. L. Knight R. Bradford M. A. Fierer N. (2010). Consistent effects of nitrogen fertilization on soil bacterial communities in contrasting systems. *Ecology* 91 3463–3470 10.1890/10-0426.1 21302816

[B55] Roesch L. F. W. Fulthorpe R. R. Riva A. Casella G. Hadwin A. K. M. Kent A. D. (2007). Pyrosequencing enumerates and contrasts soil microbial diversity. *ISME J.* 1 283–290. 18043639 10.1038/ismej.2007.53 PMC2970868

[B56] Rousk J. Baath E. Brookes P. C. Lauber C. L. Lozupone C. Caporaso J. G. (2010). Soil bacterial and fungal communities across a pH gradient in an arable soil. *ISME J.* 4 1340–1351 10.1038/ismej.2010.58 20445636

[B57] Sait M. Davis K. E. Janssen P. H. (2006). Effect of pH on isolation and distribution of members of subdivision 1 of the phylum *Acidobacteria* occurring in soil. *Appl. Environ. Microbiol.* 72 1852–1857 10.1128/AEM.72.3.1852-1857.2006 16517631 PMC1393200

[B58] Sait M. Hugenholtz P. Janssen P. H. (2002). Cultivation of globally distributed soil bacteria from phylogenetic lineages previously only detected in cultivation-independent surveys. *Environ. Microbiol.* 4 654–666 10.1046/j.1462-2920.2002.00352.x 12460273

[B59] Schaberg P. Minocha R. Long S. Halman J. Hawley G. Eagar C. (2011). Calcium addition at the Hubbard Brook experimental forest increases the capacity for stress tolerance and carbon capture in red spruce (*Picea rubens*) trees during the cold season. *Trees* 25 1053–1061 10.1007/s00468-011-0580-8

[B60] Shannon C. E. (1948). A mathematical theory of communication. *Bell Syst. Tech. J.* 27 379–423 10.1002/j.1538-7305.1948.tb01338.x

[B61] Shortle W. C. Smith K. T. (1988). Aluminum-induced calcium deficiency syndrome in declining red spruce. *Science* 240 1017–1018 10.1126/science.240.4855.1017 17731713

[B62] Simon C. Daniel R. (2011). Metagenomic analyses: past and future trends. *Appl. Environ. Microbiol.* 77 1153–1161 10.1128/AEM.02345-10 21169428 PMC3067235

[B63] Sogin M. L. Morrison H. G. Huber J. A. Welch D. M. Huse S. M. Neal P. R. (2006). Microbial diversity in the deep sea and the underexplored “rare biosphere”. *Proc. Natl. Acad. Sci. U.S.A.* 103 12115–12120 10.1073/pnas.0605127103 16880384 PMC1524930

[B64] Sridevi G. Minocha R. Turlapati S. A. Goldfarb K. C. Brodie E. L. Tisa L. S. (2012). Soil bacterial communities of a calcium-supplemented and a reference watershed at the Hubbard Brook Experimental Forest (HBEF), New Hampshire, USA. *FEMS Microbiol. Ecol.* 79 728–740 10.1111/j.1574-6941.2011.01258.x 22098093

[B65] Teeling H. Glöckner F. O. (2012). Current opportunities and challenges in microbial metagenome analysis—a bioinformatic perspective. *Brief. Bioinform.* 13 728–742 10.1093/bib/bbs039 22966151 PMC3504927

[B66] Torsvik V. Øvreås L. Thingstad T. F. (2002). Prokaryotic diversity–magnitude, dynamics, and controlling factors. *Science* 296 1064–1066 10.1126/science.1071698 12004116

[B67] Treseder K. K. (2008). Nitrogen additions and microbial biomass: a meta-analysis of ecosystem studies. *Ecol. Lett.* 11 1111–1120 10.1111/j.1461-0248.2008.01230.x 18673384

[B68] Trevors J. T. (2010). One gram of soil: a microbial biochemical gene library. *Antonie Van Leeuwenhoek* 97 99–106 10.1007/s10482-009-9397-5 19921459

[B69] Turlapati S. A. Minocha R. Bhiravarasa P. S. Tisa L. S. Thomas W. K. Minocha S. C. (2013). Chronic N-amended soils exhibit an altered bacterial community structure in Harvard Forest, MA, USA. *FEMS Microbiol. Ecol.* 83 478–493 10.1111/1574-6941.12009 22974374

[B70] Uroz S. Buée M. Murat C. Frey-Klett P. Martin F. (2010). Pyrosequencing reveals a contrasted bacterial diversity between oak rhizosphere and surrounding soil. *Environ. Microbiol. Rep.* 2 281–288 10.1111/j.1758-2229.2009.00117.x 23766079

[B71] VanInsberghe D. Hartmann M. Stewart G. R. Mohn W. W. (2013). Isolation of a substantial proportion of forest soil bacterial communities detected via pyrotag sequencing. *Appl. Environ. Microbiol.* 79 2096–2098 10.1128/AEM.03112-12 23315727 PMC3592214

[B72] Vartoukian S. R. Palmer R. M. Wade W. G. (2010). Strategies for culture of ‘unculturable’ bacteria. *FEMS Microbiol. Lett.* 309 1–7 10.1111/j.1574-6968.2010.02000.x. 20487025

[B73] Větrovský T. Baldrian P. (2013). The variability of the 16S rRNA gene in bacterial genomes and its consequences for bacterial community analyses. *PLoS ONE* 8:e57923 10.1371/journal.pone.0057923 PMC3583900 23460914

[B74] Wagg C. Bender S. F. Widmer F. Van Der Heijden M. G. A. (2014). Soil biodiversity and soil community composition determine ecosystem multifunctionality. *Proc. Natl. Acad. Sci. U.S.A.* 111 5266–5270 10.1073/pnas.1320054111 24639507 PMC3986181

[B75] Wall D. H. Bardgett R. D. Kelly E. (2010). Biodiversity in the dark. *Nat. Geosci.* 3 297–298 10.1038/ngeo860

[B76] Wallenstein M. D. Mcnulty S. Fernandez I. J. Boggs J. Schlesinger W. H. (2006). Nitrogen fertilization decreases forest soil fungal and bacterial biomass in three long-term experiments. *For. Ecol. Manage.* 222 459–468 10.1016/j.foreco.2005.11.002

[B77] Wertz S. Leigh A. K. Grayston S. J. (2012). Effects of long-term fertilization of forest soils on potential nitrification and on the abundance and community structure of ammonia oxidizers and nitrite oxidizers. *FEMS Microbiol. Ecol.* 79 142–154 10.1111/j.1574-6941.2011.01204.x 22066501

[B78] Wu M. Qin H. Chen Z. Wu J. Wei W. (2011). Effect of long-term fertilization on bacterial composition in rice paddy soil. *Biol. Fertil. Soils* 47 397–405 10.1007/s00374-010-0535-z

[B79] Wu T. Chellemi D. O. Graham J. H. Martin K. J. Rosskopf E. N. (2008). Comparison of soil bacterial communities under diverse agricultural land management and crop production practices. *Microb. Ecol.* 55 293–310 10.1007/s00248-007-9276-4 17619214

[B80] Zaremba-Niedzwiedzka K. Viklund J. Zhao W. Ast J. Sczyrba A. Woyke T. (2013). Single-cell genomics reveal low recombination frequencies in freshwater bacteria of the SAR11 clade. *Genome Biol.* 14 R130. 10.1186/gb-2013-14-11-r130 PMC4053759 24286338

[B81] Zhao J. Wan S. Fu S. Wang X. Wang M. Liang C. (2013). Effects of understory removal and nitrogen fertilization on soil microbial communities in *Eucalyptus plantations*. *For. Ecol. Manage.* 310 80–86 10.1016/j.foreco.2013.08.013

