# Supplementary material for: Oligotyping reveals stronger relationship of organic soil bacterial community structure with N-amendments and soil chemistry in comparison to that of mineral soil at Harvard Forest, MA, USA
Source: Front Microbiol. 2015 Feb 16;6:49. doi: 10.3389/fmicb.2015.00049 (PMC4329816; doi:10.3389/fmicb.2015.00049)
Supplement: Supplementary file 1 [file Presentation_1.ZIP › Supplementary Materials/Suppl. Material - 1.DOCX]

#!/usr/bin/perl

#This script filters the IDs of sequences belonging to a specific phylum or class or any other hierarchical level.

#Users have to change the search term given in match_parameters accordingly.

use strict();

use warnings();

my $input_file = "usga_classified.txt";

my $match_parameters = "Acidobacteria";

my $output_file = "Acidobacteria_output.txt";

open(FILE, $input_file) || die;

my @lines = <FILE>;

my $output;

for (@lines)

{

if ($_ =~ m/$match_parameters/)

{

$output .= $_;

}

}

close FILE;

open(OUTPUT, ">$output_file");

print OUTPUT $output;

close OUTPUT;
