# Supplementary material for: Oligotyping reveals stronger relationship of organic soil bacterial community structure with N-amendments and soil chemistry in comparison to that of mineral soil at Harvard Forest, MA, USA
Source: Front Microbiol. 2015 Feb 16;6:49. doi: 10.3389/fmicb.2015.00049 (PMC4329816; doi:10.3389/fmicb.2015.00049)
Supplement: Supplementary file 1 [file Presentation_1.ZIP › Supplementary Materials/Suppl. Matierial - 2.DOCX]

#This script filters OTUs that occur in less than 2 samples.

#This script also filters OTUs based on abundance and the user has to define the number of sequences to retain a OTU.

import sys

import re

input_file = sys.argv[1]

output_file = sys.argv[2]

line_cutoff = sys.argv[3]

output = ""

with open(input_file, 'r') as f:

w = open(output_file, 'w')

for line in f:

elements = line.split("\t")

elements.pop(0)

for idx, i in enumerate(elements):

elements[idx] = re.sub(r'Min\_\d*', "Min", elements[idx])

elements[idx] = re.sub(r'Org\_\d*', "Org", elements[idx])

elements[idx] = elements[idx].rstrip()

if len(elements) >= int(line_cutoff):

if len(set([x for x in elements if elements.count(x) > 1])) == 0:

output += line

elif len(elements) > 2:

#Check to see if there are at least some different elements

dif_counter = 0

for i in set([x for x in elements if elements.count(x) > 1]):

if elements.count(i) > 1: dif_counter += elements.count(i);

if len(elements) != dif_counter:

output += line

w.write(output)

w.close()

# for idx, i in enumerate(elements):

# elements[idx].sub(r'Min\_\d*', "Min", elements[idx])

# elements[idx].sub(r'Org\_\d*', "Org", elements[idx])

pass
