# Supplementary material for: Oligotyping reveals stronger relationship of organic soil bacterial community structure with N-amendments and soil chemistry in comparison to that of mineral soil at Harvard Forest, MA, USA
Source: Front Microbiol. 2015 Feb 16;6:49. doi: 10.3389/fmicb.2015.00049 (PMC4329816; doi:10.3389/fmicb.2015.00049)
Supplement: Supplementary file 1 [file Presentation_1.ZIP › Supplementary Materials/Suppl. Table 1.DOCX]

| **Sample Id** | **Sequences after Quality Filtered in QIIME** | **Chimeric Sequences** | **Sequences for oligotyping after quality**  **filtering and chimera removal** |
| --- | --- | --- | --- |
| **ConOrg-A5** | 17072 | 449 | 16623 |
| **ConOrg-B2** | 14640 | 258 | 14382 |
| **ConOrg-C3** | 12728 | 62 | 12666 |
| **ConOrgD4** | 17952 | 597 | 17355 |
| **ConOrg-E1** | 15859 | 222 | 15637 |
| **LN-Org-A3** | 21454 | 416 | 21038 |
| **LN-Org-B3** | 22220 | 368 | 21852 |
| **LN-Org-C5** | 12192 | 320 | 11872 |
| **LN-Org-E4** | 18039 | 418 | 17621 |
| **LN-Org-F2** | 24867 | 628 | 24239 |
| **HNOrg-A4** | 18984 | 703 | 18281 |
| **HNOrg-B6** | 20018 | 446 | 19572 |
| **HNOrg-D6** | 17399 | 363 | 17036 |
| **HNOrg-E2** | 21730 | 581 | 21149 |
| **HNOrg-E4** | 21539 | 458 | 21081 |
| **ConMin-A5** | 15519 | 168 | 15351 |
| **ConMin-B2** | 13396 | 193 | 13203 |
| **ConMin-D4** | 16447 | 312 | 16135 |
| **ConMin-C4** | 18665 | 410 | 18255 |
| **ConMin-E1** | 15239 | 170 | 15069 |
| **LNMin-A3** | 14138 | 98 | 14040 |
| **LNMin-B3** | 15830 | 151 | 15679 |
| **LNMin-C5** | 18819 | 209 | 18610 |
| **LNMin-E4** | 26896 | 312 | 26584 |
| **LNMin-F2** | 19037 | 426 | 18611 |
| **HNMin-A4** | 18046 | 306 | 17740 |
| **HNMin-B6** | 16706 | 222 | 16484 |
| **HNMin-D6** | 15947 | 210 | 15737 |
| **HNMin-E2** | 13297 | 216 | 13081 |
| **HNMin-E4** | 12665 | 120 | 12545 |
|  |  |  |  |
| **Total seqs** | **527340** | **9812** | **517528** |
| **Mean seq** | **17578** | **327** | **17251** |
| **SE** | **658** | **30** | **639** |

**Suppl. Table 1.** Raw sequence data after quality filtering and chimera removal for each of the 30 soil samples (modified from Turlapati et al. 2013).
