# Supplementary material for: Oligotyping reveals stronger relationship of organic soil bacterial community structure with N-amendments and soil chemistry in comparison to that of mineral soil at Harvard Forest, MA, USA
Source: Front Microbiol. 2015 Feb 16;6:49. doi: 10.3389/fmicb.2015.00049 (PMC4329816; doi:10.3389/fmicb.2015.00049)
Supplement: Supplementary file 1 [file Presentation_1.ZIP › Supplementary Materials/Suppl. Table 2.DOCX]

**Suppl. Table 2.** Details on oligotyping analyses of 16S rRNA sequence data for 30 soil samples. QF = quality filtering with s and M.

| **Phylum** | **Subgroup**  **Or Class** | **Total No. of RDP**  **classified seqs at CT >0.8** | **% of total sequences** | **No. of seqs after QF* in oligotyping**  **(% of total)** | **No. of entropy components**  **chosen** | **M value** | **No. of oligotypes** | **% of total oligotypes** | **Genera at CT >0.8** | **GenBank**  **Accession numbers**  **(NCBI)** |
| --- | --- | --- | --- | --- | --- | --- | --- | --- | --- | --- |
| *Acidobacteria* | *Gp1* | 151,462 | 27.6 | 132,062 (87%) | 100 | 50 | 295 | 6.5 | 3 | KP010414 - KP010709 |
| *Acidobacteria* | *Gp2* | 67,374 | 12.3 | 61,688 (92%) | 69 | 25 | 193 | 4.3 | 0 | KP076667 - KP076859 |
| *Acidobacteria* | *Gp3* | 45,196 | 8.2 | 36,758 (81% ) | 85 | 20 | 300 | 6.6 | 1 | KP076860 - KP077176 |
| *Acidobacteria* | *Gp4* | 715 | 0.1 | 618 (86%) | 55 | 5 | 13 | 0.3 | 0 | KP077177 - KP077191 |
| *Acidobacteria* | *Gp5* | 1,808 | 0.3 | 1,808 (82%) | 74 | 5 | 42 | 0.9 | 0 | KP077192 - KP077234 |
| *Acidobacteria* | *Gp6* | 4,979 | 0.9 | 4,343 (87%) | 46 | 10 | 52 | 1.2 | 0 | KP077235 - KP077289 |
| *Acidobacteria* | *Gp7* | 1,138 | 0.2 | 1,009 (89%) | 22 | 5 | 24 | 0.5 | 0 | KP077290 - KP077313 |
| *Acidobacteria* | *Gp10* | 1,684 | 0.3 | 1,599 (95%) | 18 | 5 | 14 | 0.3 | 0 | KP077314 - KP077326 |
| *Acidobacteria* | *Gp13* | 1,851 | 0.3 | 1,618 (87%) | 51 | 5 | 54 | 1.2 | 0 | KP077327 - KP077383 |
| ***Acidobacteria*** | ***Total*** | **276,207** | **50** |  |  |  | **987** | **22** | **4** |  |
| *Proteobacteria* | *α-Proteo* | 38,858 | 7.1 | 26,073 (67%) | 69 | 15 | 389 | 8.6 | 11 | KP077384 - KP077772 |
| *Proteobacteria* | *β-Proteo* | 3,340 | 0.6 | 2,887 (86%) | 54 | 3 | 123 | 2.7 | 8 | KP077773 - KP077895 |
| *Proteobacteria* | *δ-Proteo* | 24,560 | 4.5 | 16,694 (68%) | 93 | 10 | 134 | 3.0 | 0 | KP077896 - KP078226 |
| *Proteobacteria* | *γ-Proteo* | 41,745 | 7.6 | 30,777 (74%) | 52 | 15 | 287 | 6.3 | 11 | KP078227 - KP078513 |
| ***Proteobacteria*** | ***Total*** | **108,503** | **20** |  |  |  | **933** | **21** | **30** |  |
| *Actinobacteria* |  | 39,904 | 7.3 | 28,699 (72%) | 103 | 15 | 343 | 7.6 | 11 | KP078514 - KP078867 |
| *Verrucomicrobia* |  | 55,270 | 10.1 | 44,715 (81%) | 115 | 15 | 452 | 10.0 | 2 | KP078868 - KP079321 |
| *Chlamydiae* |  | 13,928 | 2.5 | 9,311 (67%) | 83 | 5 | 463 | 10.2 | 3 | KP079322 - KP079789 |
| *Chloroflexi* |  | 14,564 | 2.7 | 11,364 (78%) | 90 | 5 | 293 | 6.5 | 2 | KP079790 - KP080169 |
| *Cyanobacteria* |  | 4,121 | 0.8 | 3,138 (76%) | 92 | 3 | 280 | 6.2 | 0 | KP080170 - KP080449 |
| *Elusimicrobia* |  | 4,252 | 0.8 | 2,802 (66%) | 77 | 5 | 197 | 4.3 | 1 | KP080450 - KP080646 |
| *Firmicutes* |  | 1,686 | 0.3 | 1,063 (63%) | 70 | 2 | 137 | 3.0 | 12 | KP080647 - KP080783 |
| *Gemmatimonadetes* |  | 3,514 | 0.6 | 3,210 (91%) | 45 | 5 | 14 | 0.3 | 1 | KP080784 - KP080851 |
| TM7 |  | 1,110 | 0.2 | 576 (52%) | 97 | 2 | 91 | 2.0 | 0 | KP080852 - KP080958 |
| WPS-2 |  | 10,643 | 1.9 | 9,768 (92%) | 64 | 5 | 161 | 3.6 | 0 | KP080959 - KP081119 |
| AD3 |  | 9,217 | 1.7 | 8,444 (92%) | 54 | 5 | 142 | 3.1 | 0 | KP081120 - KP081261 |
| *Bacteroidetes* |  | 259 | 0.1 | 92 (36%) | 49 | 2 | 36 | 0.8 | 6 | KP081262 - KP081297 |
| *Nitrospira* |  | 661 | 0.1 | 616 (93%) | 57 | 2 | 5 | 0.1 | 1 | KP081298 - KP081313 |
| TM6 |  | 36 | 0.0 | - | - | - | - | - | 0 |  |
| **Unclassified** |  | **5,625** | 1.0 |  |  |  |  |  |  |  |
| **Total** |  | **549,500** | **100.0** |  |  |  | **4534** | **100.0** | **73** |  |
