# Supplementary material for: Oligotyping reveals stronger relationship of organic soil bacterial community structure with N-amendments and soil chemistry in comparison to that of mineral soil at Harvard Forest, MA, USA
Source: Front Microbiol. 2015 Feb 16;6:49. doi: 10.3389/fmicb.2015.00049 (PMC4329816; doi:10.3389/fmicb.2015.00049)
Supplement: Supplementary file 1 [file Presentation_1.ZIP › Supplementary Materials/Suppl. Table 3.DOCX]

**Suppl. Table 3.** Soil chemistry of control and N–amended soils from organic and mineral horizons. Data are mean ± SE of 5 replicate soil samples for each treatment. **^a^** (*P* ≤0.05) and **^b^** (*P* ≤0.10) denote significant differences between the organic and mineral soil horizons of respective treatment. **^**^** (*P* ≤0.05) and **^*^** (*P* ≤0.10) denote significant differences between the control and N-amended treatments. Data were used for Mantel tests of each individual phylum using oligotyping data. Except for the data shown in bold, rest of the data were published in our previous reports ([Turlapati et al. 2013](#_ENREF_2)) and ([Frey et al. 2014](#_ENREF_1)).

|  | **Organic Soil Horizon** | | | **Mineral Soil Horizon** | | |
| --- | --- | --- | --- | --- | --- | --- |
|  | **Control** | **Low N** | **High N** | **Control** | **Low N** | **High N** |
| **Soil pH** | 4.00 ± 0.10 | 4.10 ± 0.10 | 4.00 ± 0.15 | 4.60 ± 0.02**^a^** | 4.70 ± 0.07**^a^** | 4.60 ± 0.09**^a^** |
| **% LOI (organic matter)** | 28.1 ± 4.6 | 28.7 ± 4.6 | 27.2 ± 3.8 | 9.3 ± 0.4**^a^** | 9.5 ± 0.8**^a^** | 11.2 ± 0.4**^a^** |
| **Total N (%)** | 0.60 ± 0.08 | 0.60 ± 0.04 | 0.60 ± 0.07 | 0.20 ± 0.01**^a^** | 0.20 ± 0.02**^a^** | 0.20 ± 0.01**^a^** |
| **Total C (%)** | 15.1 ± 2.1 | 15.4 ± 2.3 | 15.0 ± 1.9 | 4.30 ± 0.2**^a^** | 4.20 ± 0.40**^a^** | 5.00 ± 0.4**^a^** |
| **Ca (mg Kg^-1^)** | 115 ± 30 | 110 ± 12 | 134 ± 37 | 13.0 ± 2.4**^a^** | 16.2 ± 2.0**^a^** | 12.6 ± 3.0**^a^** |
| **K (mg Kg^-1^)** | 223 ± 54 | 132 ± 5 | 118 ± 15**^**^** | 38.7 ± 9.0**^b^** | 37.7 ± 4.0**^a^** | 36.3 ± 4.0**^a^** |
| **Mg (mg Kg^-1^)** | 102.5 ± 29.1 | 62.3 ± 6.7 | 53.6 ± 12.6 | 11.6 ± 1.9**^a^** | 10.8 ± 1.9**^a^** | 12.0 ± 1.1**^a^** |
| **P (mg Kg^-1^)** | 24.6 ± 8.1 | 10.1 ± 2.0 | 11.5 ± 2.2 | 3.30 ± 0.6**^a^** | 1.60 ± 0.80**^a^** | 2.60 ± 0.20**^a^** |
| **Al (mg Kg^-1^)** | 513 ± 28 | 650 ± 73 | 595 ± 35 | 265 ± 29**^a^** | 224 ± 42**^a^** | 333 ± 32**^a^** |
| **Fe (mg Kg^-1^)** | 85.1 ± 7.3 | 66.5 ± 9.1 | 70.5 ± 10.9 | 12.7 ± 1.7**^a^** | 7.8 ± 4.0**^a^** | 19.0 ± 5.9**^a^** |
| **Mn (mg Kg^-1^)** | 26.8 ± 8.1 | 11.2 ± 2.1 | 13.5 ± 3.4 | 5.80 ± 2.50**^b^** | 3.60 ± 1.90**^b^** | 2.70 ± 1.70**^a^** |
| **Na (mg Kg^-1^)** | 18.9 ± 2.3 | 10.6 ± 1.9**^**^** | 14.7 ± 2.5 | 6.00 ± 0.60**^a^** | 4.10 ± 0.70**^*a^** | 7.90 ± 1.10**^a^** |
| **Zn (mg Kg^-1^)** | 17.80 ± 4.36 | 9.40 ± 1.26 | 7.60 ± 1.04**^**^** | 2.10 ± 0.69**^a^** | 2.00 ± 0.24**^a^** | 1.80 ± 0.28**^a^** |
| **Acidity (meq 100 g^-1^)** | 9.80 ± 1.10 | 9.30 ± 1.06 | 11.10 ± 0.98 | 4.00 ± 0.28**^a^** | 3.40 ± 0.64**^a^** | 4.90 ± 0.56**^a^** |
| **C:N Ratio** | 24.7 ± 0.6 | 26.3 ± 2.3 | 23.7 ± 1.9 | 21.0 ± 1.6 | 20.8 ± 1.6 | 21.1 ± 1.6 |
| **N0_3_-N (ppm)** | **0.55 ± 0.13** | **0.50 ± 0.06** | **1.09 ± 0.29** | **0.45 ± 0.09** | **0.39 ± 0.03** | **0.66 ± 0.13** |
| **NH_4_-N (ppm)** | **34.41 ± 7.64** | **31.78 ± 3.55** | **30.68 ± 5.79** | **9.87 ± 0.76^a^** | **10.82 ± 1.18^a^** | **12.70 ± 0.91^a^** |
| **Soil organic N-containing metabolites** | | | | | | |
| **Putrescine** | 16.8 ± 3.8 | 12.6 ± 2.9 | 7.6 ± 0.6* | 3.6 ± 1.1 **^a^** | 3.1 ± 0.7 **^a^** | 5.4 ± 0.8 **^a^** |
| **Spermidine** | 25.7 ± 3.7 | 18.7 ± 1.6 | 13.3 ± 1.1****** | 6.3 ± 0.7 **^a^** | 6.5 ± 0.8 **^a^** | 5.1 ± 0.9 **^a^** |
| **Glutamine** | 77.9 ± 48.4 | 185.7 ± 47.7 | 199.1 ± 64.5 | 22.5 ± 22.5 **^a^** | 53.1 ± 53.1 **^a^** | NA |
| **γ-aminobutyric acid (GABA)** | 199 ± 21.4 | 131.6 ± 6.9* | 123.3 ± 22.7** | 23.2 ± 2.7 **^a^** | 22.7 ± 4.9 **^a^** | 34.3 ± 8.2 **^a^** |
| **Alanine** | 136.6 ± 24.9 | 98.8 ± 10.2 | 70 ± 16.6* | 26.8 ± 7.2 **^a^** | 15.2 ± 1.3 **^a^** | 19.1 ± 6.5 **^a^** |
| **Glutamic acid** | **52.8 ± 5** | **55.1 ± 9.1** | **34.4 ± 11.3** | **22.1 ± 11.1^a^** | **NA** | **11.1 ± 6.8^a^** |
| **Valine** | **17.2 ± 1.7** | **11.6 ± 0.9**** | **9.7 ± 1.3**** | **5.5 ± 0.8^a^** | **4.3 ± 0.7^a^** | **4.8 ± 0.9^a^** |
| **Lysine** | **24 ± 1.8** | **18.7 ± 1.2** | **16.9 ± 2.6*** | **7.1 ± 0.6^a^** | **6.9 ± 0.8^a^** | **8.1 ± 1.2^a^** |
