# Supplementary material for: Oligotyping reveals stronger relationship of organic soil bacterial community structure with N-amendments and soil chemistry in comparison to that of mineral soil at Harvard Forest, MA, USA
Source: Front Microbiol. 2015 Feb 16;6:49. doi: 10.3389/fmicb.2015.00049 (PMC4329816; doi:10.3389/fmicb.2015.00049)
Supplement: Supplementary file 1 [file Presentation_1.ZIP › Supplementary Materials/Suppl. Table 4.DOCX]

**Suppl. Table 4.** Identification of greater number of genera at CT of ≥0.5 in comparison with CT of ≥0.8. The pyrosequencing data are from the highly diverse hypervariable region V6-V8 of 16S rRNA as compared to V4-V6, and thus is not well supported in databases ([Claesson et al. 2009](#_ENREF_1)).

|  | **RDP classifier online (RDP database)** | |
| --- | --- | --- |
| **CT for genus ID** | **80%** | **50%** |
| **Phylum** | **Number of genera** | |
| *α-Proteobacteria* | 11 | 19 |
| *β-Proteobacteria* | 8 | 13 |
| *δ-Proteobacteria* | 0 | 7 |
| *γ-Proteobacteria* | 11 | 16 |
| *Actinobacteria* | 11 | 13 |
| *Bacteroidetes* | 6 | 7 |
| *Firmicutes* | 12 | 17 |
