# Supplementary material for: Oligotyping reveals stronger relationship of organic soil bacterial community structure with N-amendments and soil chemistry in comparison to that of mineral soil at Harvard Forest, MA, USA
Source: Front Microbiol. 2015 Feb 16;6:49. doi: 10.3389/fmicb.2015.00049 (PMC4329816; doi:10.3389/fmicb.2015.00049)
Supplement: Supplementary file 1 [file Presentation_1.ZIP › Supplementary Materials/Suppl. Table 5.DOCX]

**Suppl. Table 5.** Non-metric dimensional scaling (NMS) analyses conducted using PC-ORD. Details on conditions used for NMS are described in the Materials and Methods section. To normalize the data, digit one was added to all data before log10 transformation. Bray-Curtis distances were used to delineate patterns between the various treatments and soil horizon. Dimensionality of a solution was selected based on the assessment using scree plots. Permutational MANOVA was conducted using Bray-Curtis distances to evaluate the effect of soil horizon and treatments, and the interaction between them. Non-significant correlations are shown in gray.

* denotes significant correlations at *(P ≤ 0.05).*

| **Phylum** | **Horizon (%)** | ***P*** | **Treatment (%)** | ***P*** | **NMS Solution** |
| --- | --- | --- | --- | --- | --- |
| *Acidobacteria:* | 49.0 | 0.0002* | 21.3 | 0.0004* | 2D |
| *GP1* | 50.8 | 0.0002* | 28.7 | 0.0002* | 2D |
| *GP2* | 45.6 | 0.0002* | 20.4 | 0.0008* | 2D |
| *GP3* | 50.3 | 0.0002* | 17.0 | 0.0066* | 2D |
| *GP4* | NA | NA | NA | NA | NA |
| *GP5* | 32.9 | 0.0002* | 9.0 | 0.04199* | 2D |
| *GP6* | 39.2 | 0.0002* | 7.1 | 0.0658 | 2D |
| *GP7* | NA | NA | NA | NA | NA |
| *GP10* | 7.6 | 0.0514 | 5.2 | 0.1616 | 2D |
| *GP13* | 44.6 | 0.0002* | 6.7 | 0.0226* | 3D |
| *Proteobacteria:* | 36.0 | 0.0002* | 16.5 | 0.0006* | 2D |
| *α-Proteo* | 36.7 | 0.0002* | 13.9 | 0.00279* | 2D |
| *β-Proteo* | 24.9 | 0.0002* | 8.8 | 0.003* | 3D |
| *δ-Proteo* | 27.8 | 0.0002* | 10.9 | 0.0002* | 2D |
| *γ-Proteo* | 37.8 | 0.0002* | 23.2 | 0.0002* | 2D |
| *Actinobacteria* | 44.1 | 0.0002* | 10.3 | 0.03639* | 2D |
| *Verrucomicrobia* | 44.3 | 0.0002* | 13.4 | 0.0058* | 2D |
| *Chlamydiae* | 9.6 | 0.0006* | 18.9 | 0.0002* | 3D |
| *Chloroflexi* | 23.8 | 0.0002* | 5.1 | 0.0594 | NS |
| *Cyanobacteria* | 18.6 | 0.0002* | 11.6 | 0.0002* | 3D |
| *Elusimicrobia* | 11.8 | 0.0002* | 7.9 | 0.0004* | 3D |
| *Firmicutes* | 14.7 | 0.0002* | 0.0 | 0.8076 | 3D |
| *Gemmatimonadetes* | 40.9 | 0.0002* | 12.6 | 0.04499* | 3D |
| TM7 | 12.6 | 0.0002* | 4.8 | 0.1330 | 4D |
| WPS-2 | 41.5 | 0.0002* | 16.1 | 0.0016* | 2D |
| AD3 | 34.0 | 0.0002* | 11.4 | 0.0076* | 2D |
| *Bacteroidetes* | - | 0.1958 | - | 0.2143 | 1D |
