# Supplementary material for: Oligotyping reveals stronger relationship of organic soil bacterial community structure with N-amendments and soil chemistry in comparison to that of mineral soil at Harvard Forest, MA, USA
Source: Front Microbiol. 2015 Feb 16;6:49. doi: 10.3389/fmicb.2015.00049 (PMC4329816; doi:10.3389/fmicb.2015.00049)
Supplement: Supplementary file 1 [file Presentation_1.ZIP › Supplementary Materials/Suppl. Table 6.DOCX]

**Suppl. Table 6.** Comparisons of the presence of oligotypes of the same genus in organic and mineral horizons of untreated soils. Total number of oligotypes in a genus denote all oligotypes that were found in 30 soil samples. Unique oligotypes found in the N-amended soils are the difference between the total count and the controls. While an empty cell denotes the presence of respective oligotype in the sample NA denotes absence.

| **Phylum *Verrucomicrobia* : Genus *Opitutus*** | | | **Phylum *Firmicutes :* Genus *Bacillus*** | | | **Phylum *Acidobacteria :* Genus *Edaphobacter*** | | |
| --- | --- | --- | --- | --- | --- | --- | --- | --- |
| **Oligotype ID** | **Con-Org** | **Con-Min** | **Oligotype ID** | **Con-Org** | **Con-Min** | **Oligotype ID** | **Con-Org** | **Con-Min** |
| Verru_OR311 | NA |  | Firmi_OR18 | NA |  | gp1_OR176 |  |  |
| Verru_OR392 | NA |  | Firmi_OR44 | NA |  | gp1_OR37 |  |  |
| Verru_OR46 |  |  | Firmi_OR56 | NA |  | gp1_OR54 |  |  |
| Verru_OR71 |  |  | Firmi_OR11 | NA |  | gp1_OR157 |  |  |
| Verru_OR95 |  |  | Firmi_OR85 | NA |  | gp1_OR237 |  |  |
| Verru_OR121 |  |  | Firmi_OR23 | NA |  | gp1_OR71 |  |  |
| Verru_OR54 |  |  | Firmi_OR66 | NA |  | gp1_OR101 |  |  |
| Verru_OR119 |  |  | Firmi_OR122 | NA |  | gp1_OR267 |  | NA |
| Verru_OR326 |  |  | Firmi_OR42 | NA |  | gp1_OR172 |  |  |
| Verru_OR229 |  |  | Firmi_OR36 | NA |  | gp1_OR250 |  | NA |
| Verru_OR430 |  | NA | Firmi_OR28 | NA |  | gp1_OR126 |  |  |
| Verru_OR446 |  | NA | Firmi_OR2 |  |  | gp1_OR279 |  |  |
| Verru_OR454 |  | NA | Firmi_OR13 |  |  | gp1_OR189 |  |  |
| Verru_OR189 |  |  | Firmi_OR43 |  |  | gp1_OR116 |  |  |
| Verru_OR208 |  |  | Firmi_OR7 |  |  | gp1_OR180 |  | NA |
| Verru_OR272 |  |  | Firmi_OR121 |  |  | gp1_OR69 |  | NA |
| Verru_OR351 |  | NA | Firmi_OR112 |  | NA | gp1_OR293 |  | NA |
| Verru_OR309 |  | NA | Firmi_OR67 |  | NA |  |  |  |
| Verru_OR292 |  |  | Firmi_OR84 |  | NA |  |  |  |
| Verru_OR132 |  |  |  |  |  |  |  |  |
| Verru_OR347 |  | NA |  |  |  |  |  |  |
| Verru_OR369 |  | NA |  |  |  |  |  |  |
| Verru_OR308 |  | NA |  |  |  |  |  |  |
| Verru_OR136 |  |  |  |  |  |  |  |  |
| Verru_OR393 |  |  |  |  |  |  |  |  |
| Verru_OR412 |  |  |  |  |  |  |  |  |
| Verru_OR421 |  |  |  |  |  |  |  |  |
| **Total no. of oligotypes for each Genus** | **31** | |  | **23** | |  | **22** | |
| **Total in each soil horizon (Con plots only)** | **25** | **19** |  | **8** | **16** |  | **17** | **12** |
| **Common to both horizons** | **17** | **17** |  | **5** | **5** |  | **12** | **12** |
| **Exclusive to each soil horizon** | **8** | **2** |  | **3** | **11** |  | **5** | **0** |
