# Supplementary material for: Oligotyping reveals stronger relationship of organic soil bacterial community structure with N-amendments and soil chemistry in comparison to that of mineral soil at Harvard Forest, MA, USA
Source: Front Microbiol. 2015 Feb 16;6:49. doi: 10.3389/fmicb.2015.00049 (PMC4329816; doi:10.3389/fmicb.2015.00049)
Supplement: Supplementary file 1 [file Presentation_1.ZIP › Supplementary Materials/Suppplementary Figures.DOCX]

**Supplemental Figure 1 Page 1 of 1**

**
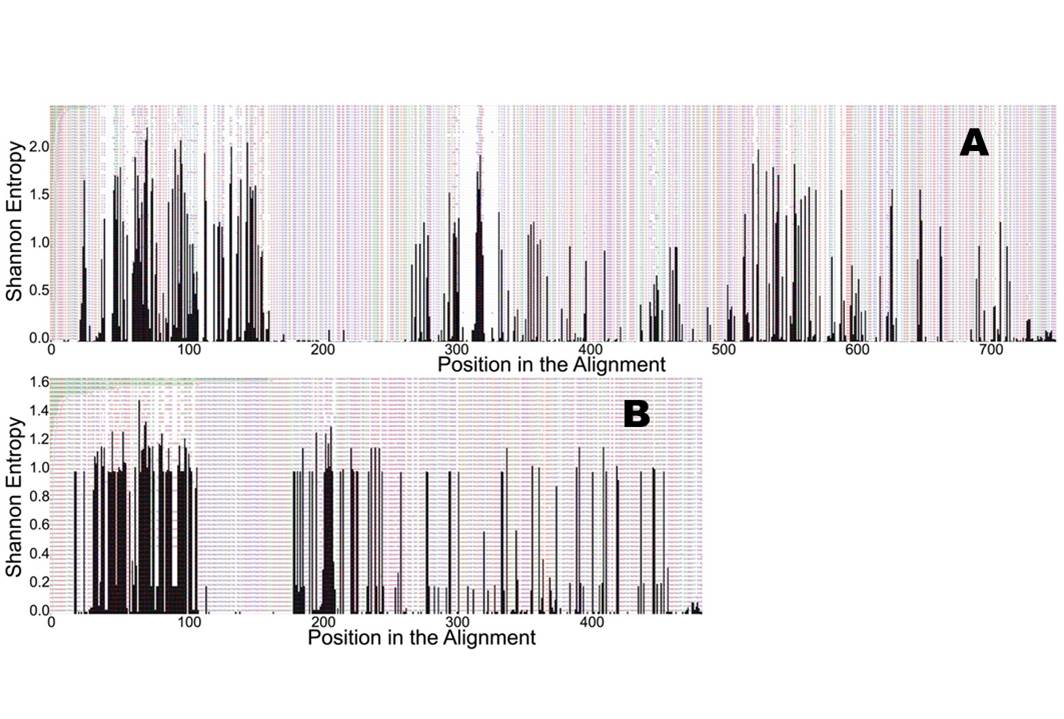
**

**Supplemental Figure 1:** Entropy analysis results on: A) class *α-Proteobacteria* of the phylum *Proteobacteria* with 38,858 sequences and 752 characters long in the alignment; B) phylum *Nitrospira* with 661 sequences and 486 characters long in the alignment. Bars show the Shannon entropy value for each position.

**Supplemental Figure 2 Page 1 of 1**

**Supplemental Figure 2:** Sequences of genus *Terriglobus* of the *Gp1* subgroup in *Acidobacteria* were found in all treatments in both soil horizons (all 30 plots in this study). This genus could not be identified at CT ≥0.8 but was discernible at CT value ≥0.5. Data presented are mean ± SE of the number of sequences corresponding to this genus from five replicate plots per treatment.

**Supplemental Figure 3 Page 1 of 3**

**Supplemental Figure 3 Page 2 of 3**

**Page 3 of 3**

**Supplemental Figure 3:** NMS ordination for oligotypes of 30 soil samples for all subgroups within *Acidobacteria* (A-G), classes within *Proteobacteria* (H-K) and the phylum TM7 (L). Each soil type is represented by five replicates and a centroid, which is indicated by a single symbol and treatment-soil horizon name. The number included in each graph title represents the total number of oligotypes identified within each taxonomic group. H = the percent of variation partitioned by horizon and T = the percent of variation partitioned by treatment. No figure was generated for *Bacteroidetes* because of one-dimensional solution.

**Supplemental Figure 4 Page 1 of 2**

**Supplemental Figure 4 Page 2 of 2**

**Supplemental Figure 4:** Partitioning of sequences (left side panels) and oligotypes (right side panels) with treatments for all identified subgroups within the phylum *Acidobacteria* present in organic (A) and mineral (B) soils. The figures depict differences in the proportions of sequences and the oligotypes.

**Supplemental Figure 5 Page 1 of 8**

**
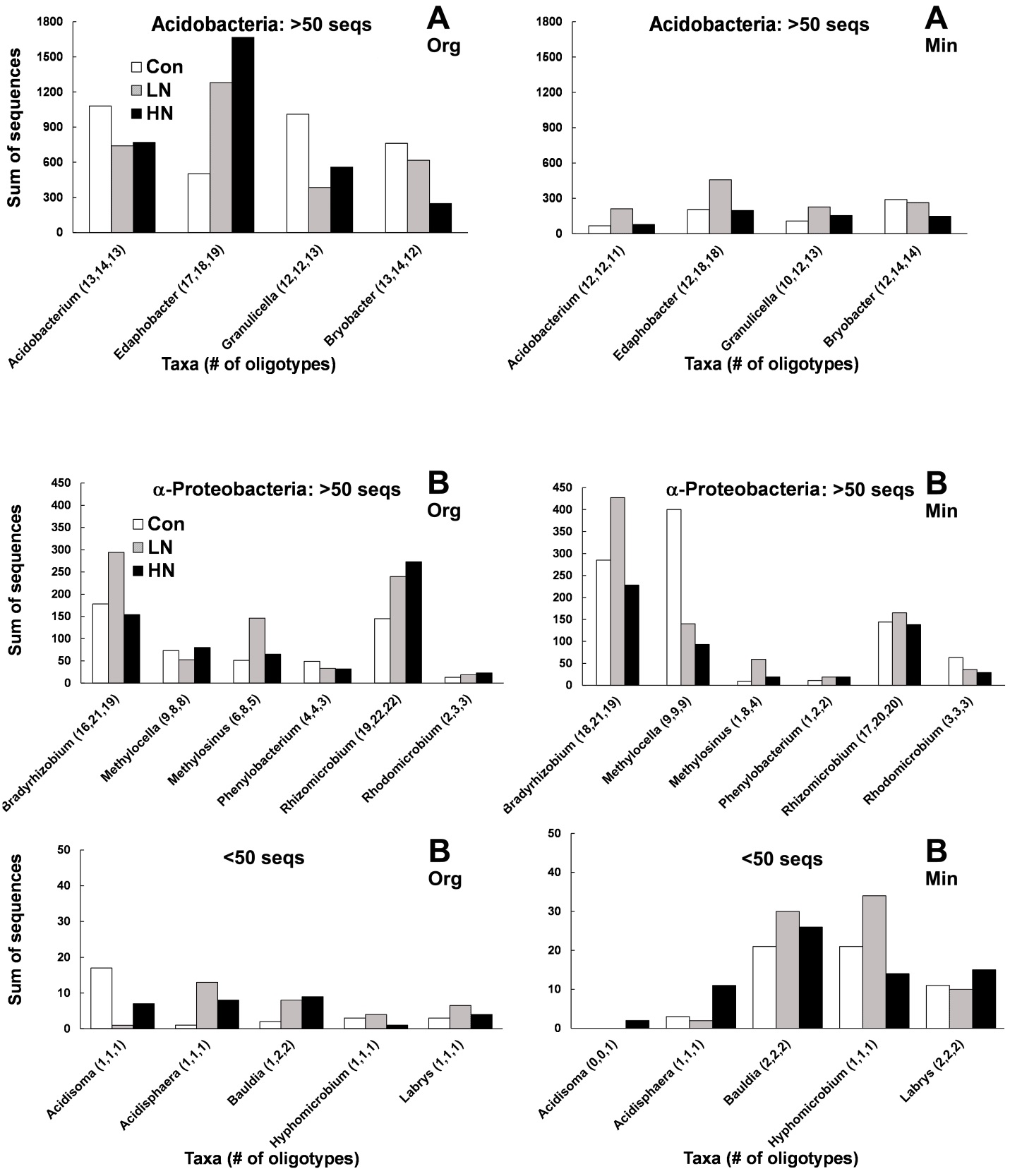
**

**Supplemental Figure 5** **Page 2 of 8**


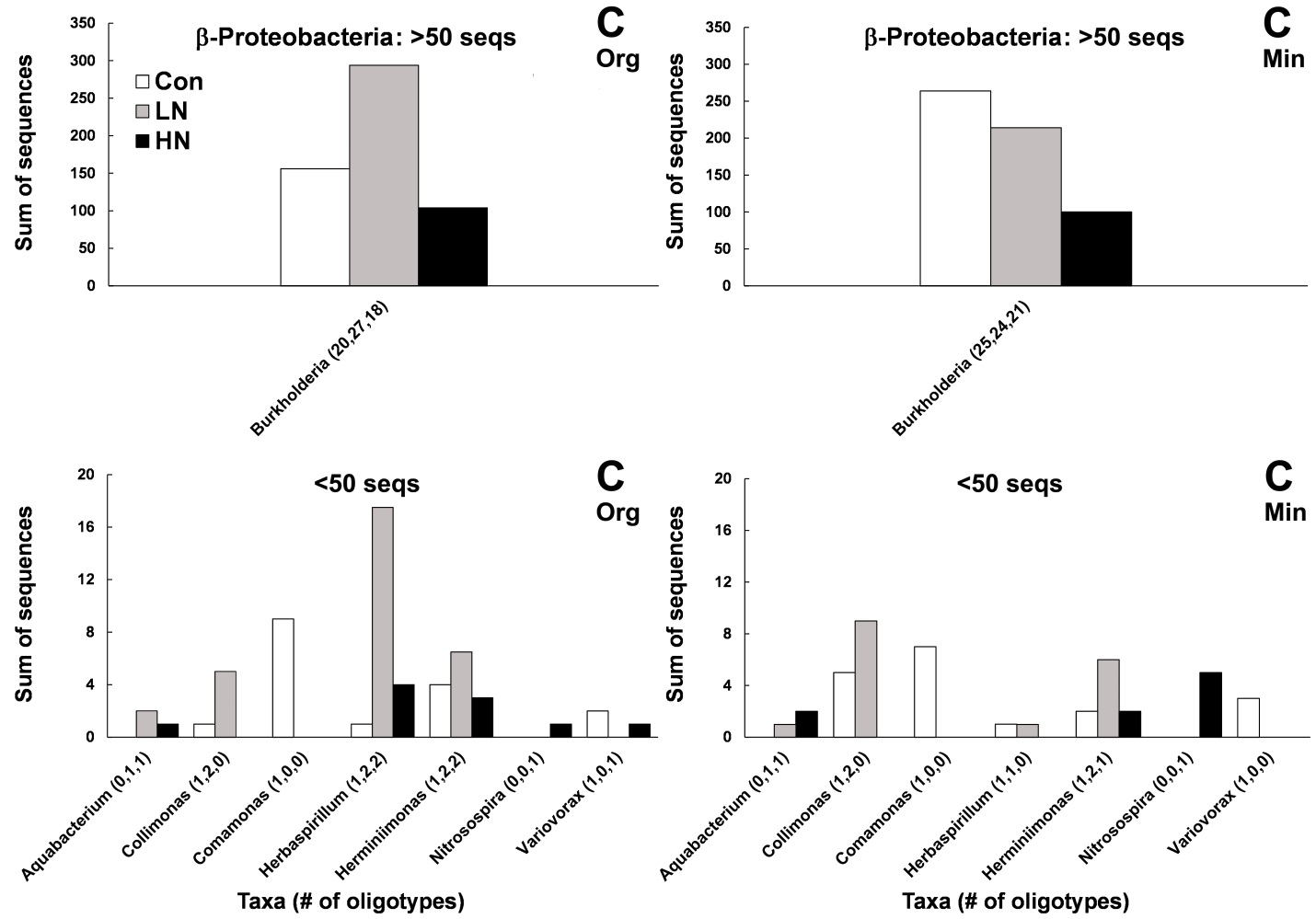


**Supplemental Figure 5** **Page 3 of 8**


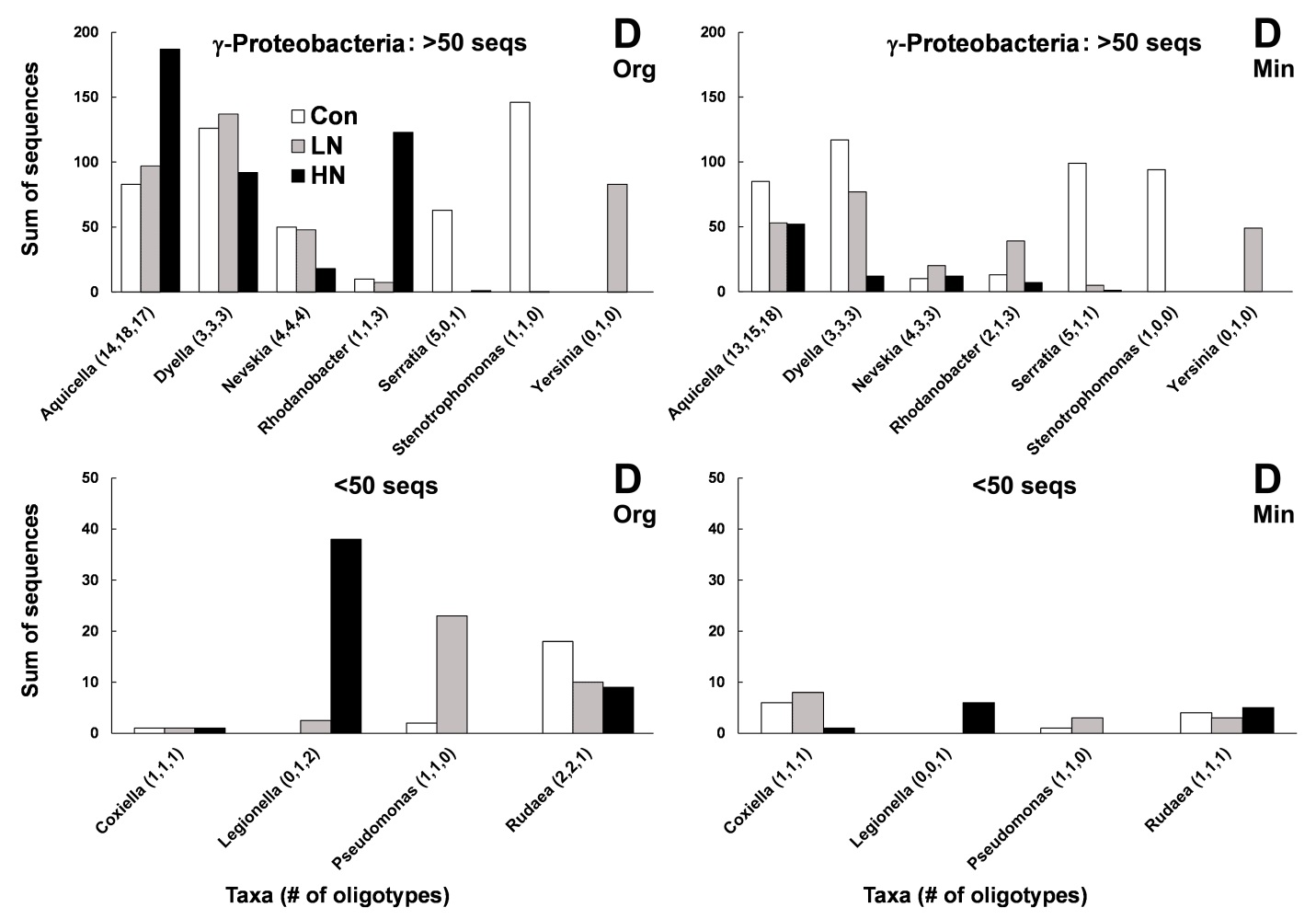


**Supplemental Figure 5 Page 4 of 8**


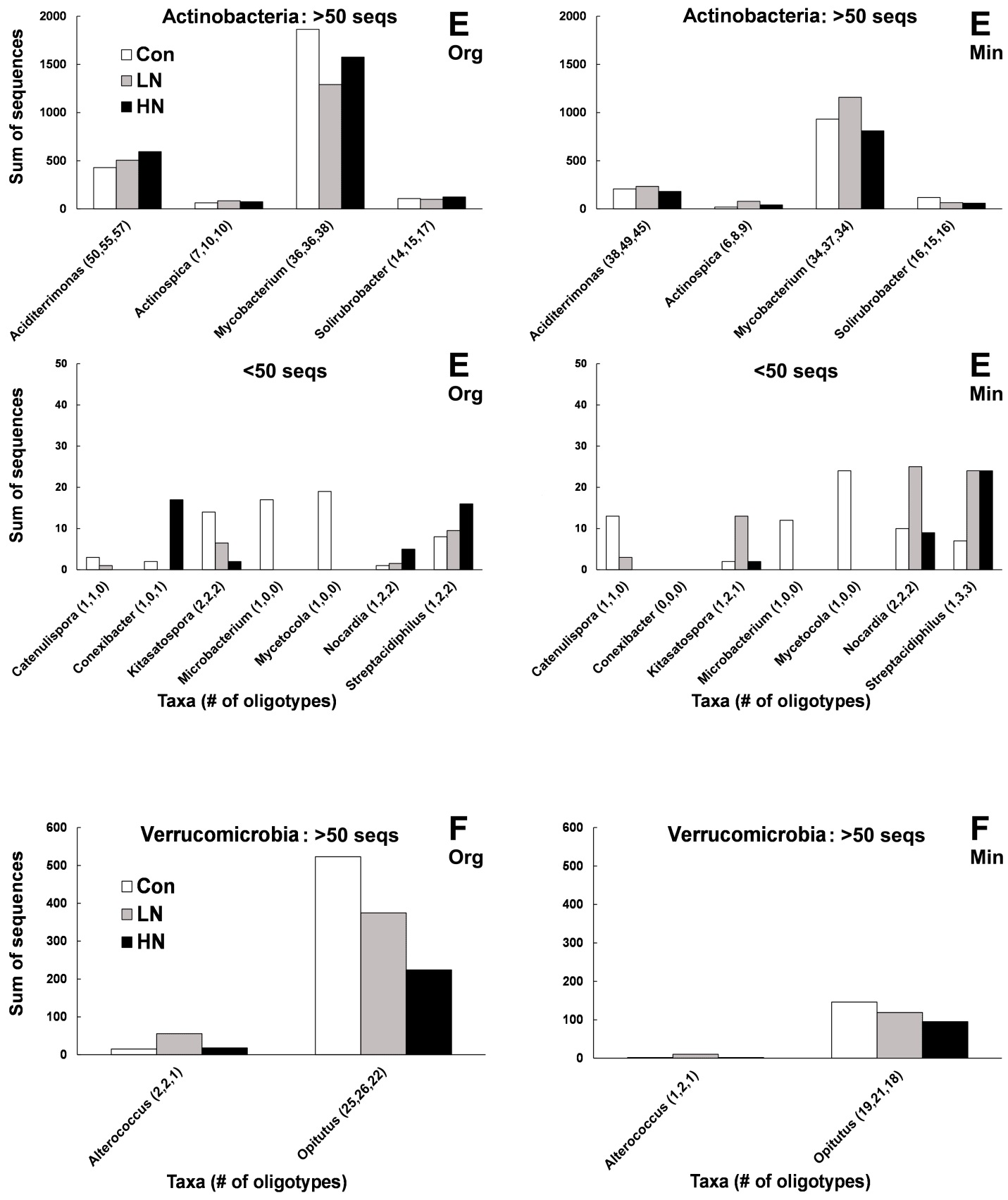


**Supplemental Figure 5 Page 5 of 8**


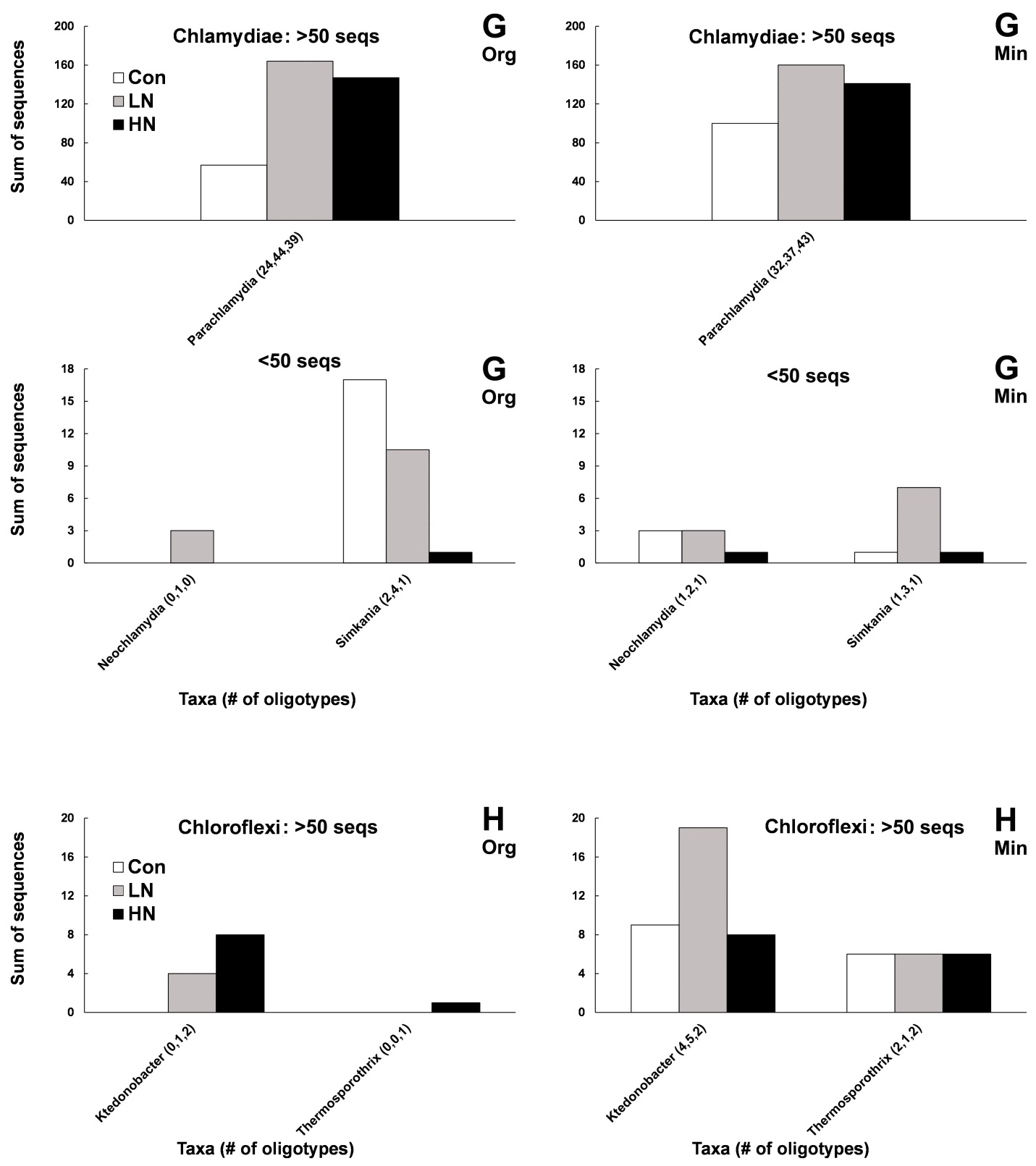


**Supplemental Figure 5 Page 6 of 8**


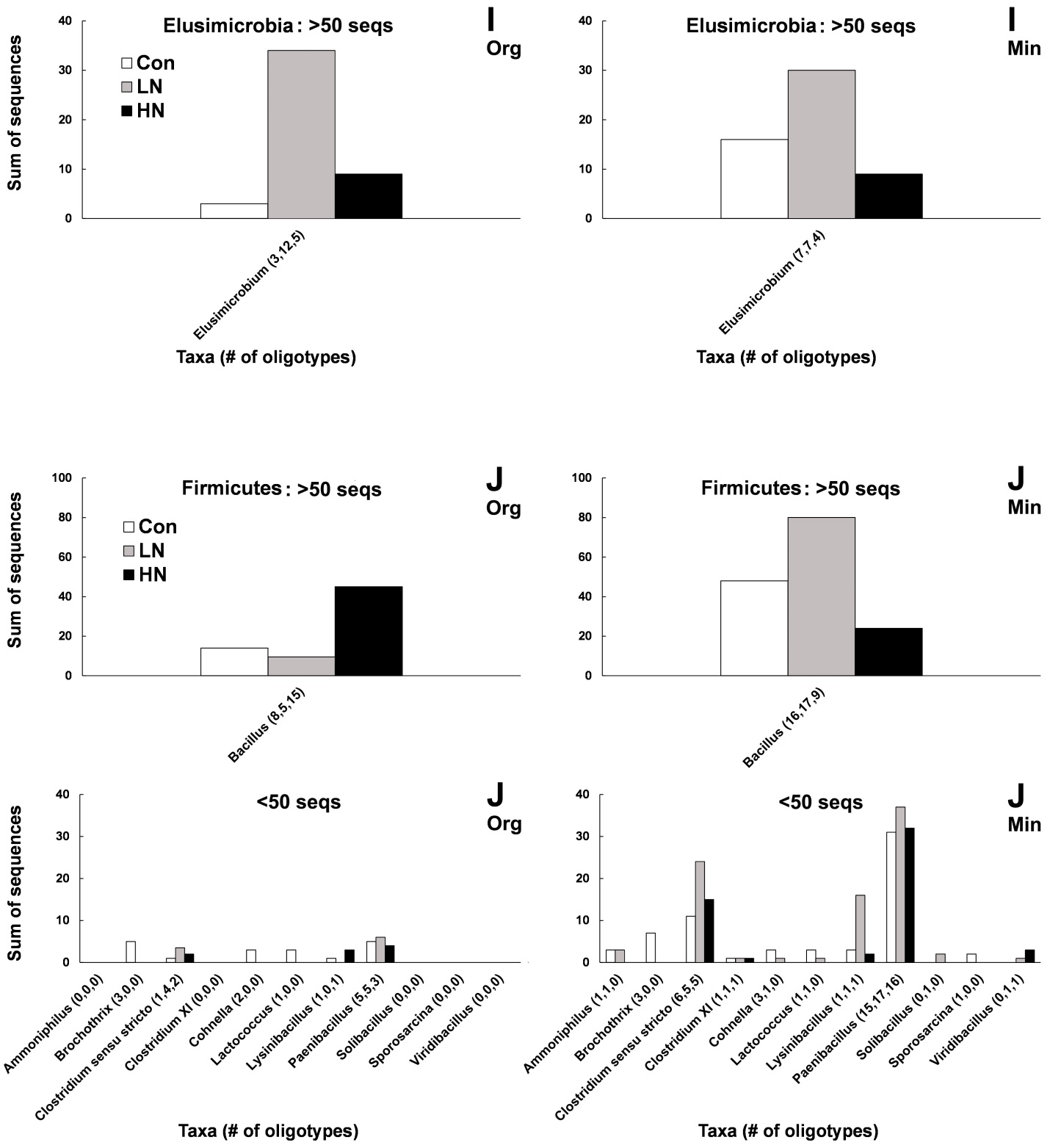


**Supplemental Figure 5 Page 7 of 8**


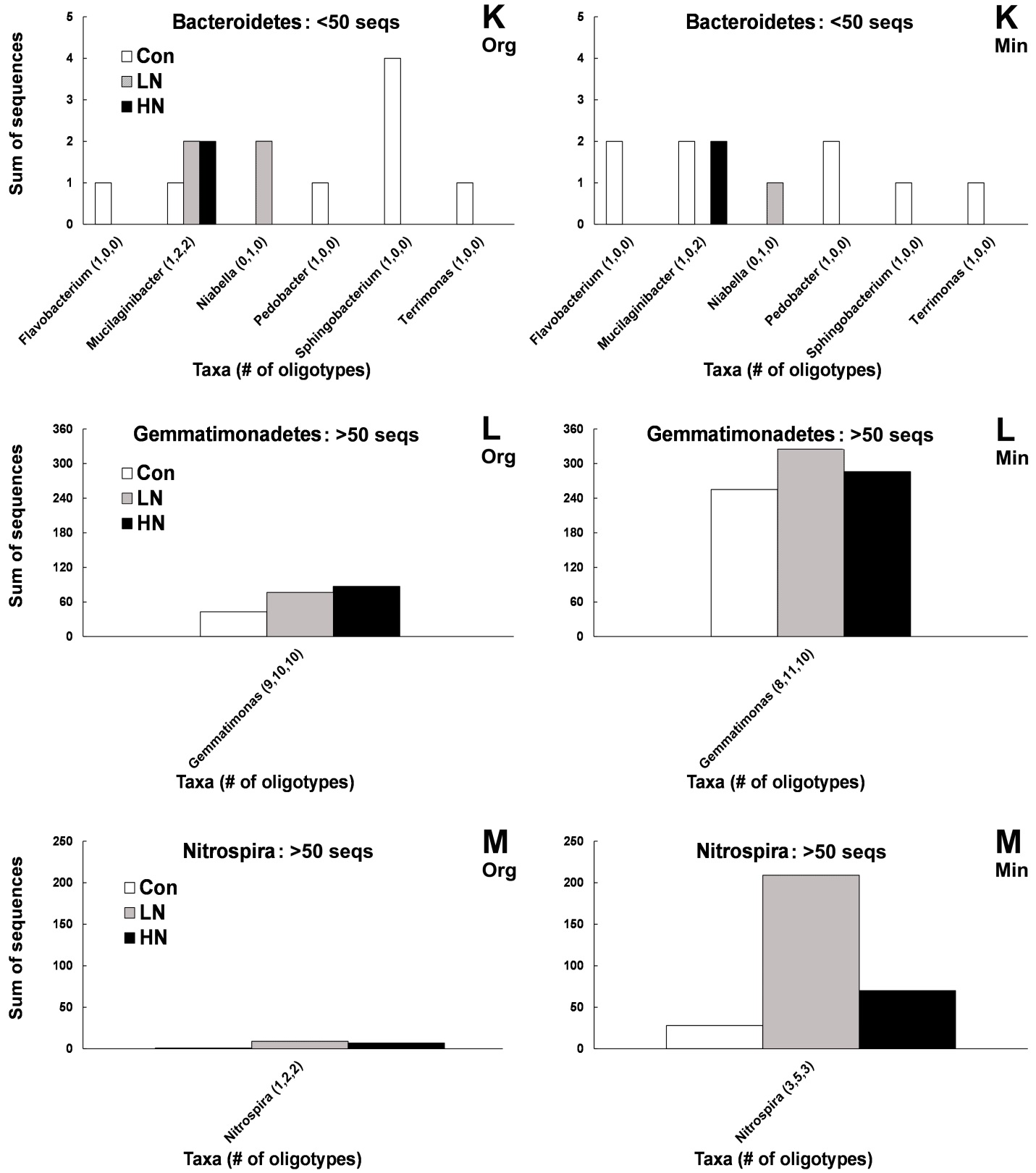


**Page 8 of 8**

**Supplemental Figure 5:** Total number of sequences with total number of oligotypes per treatment in organic and mineral soil for each genus. These figures show all genera identified from the bacterial phyla present in Harvard Forest soils. Each genus is represented by 2-4 graph panels; Genera represented by >50 sequences (combined from five replicate samples) in any of the 6 soil samples (2 horizons x 3 treatments) are presented in the top panels of the figure while low abundance genera (<50 sequences) are presented in the bottom panels. Organic soil data are presented on the left side and mineral on the right side panels of the figure.

**Supplemental Figure 6 Page 1 of 2**

**Supplemental Figure 6 Page 2 of 2**

**Supplemental Figure 6:** Partitioning of sequences (left side panels) and oligotypes (right side panels) with treatments for all identified classes within the phylum *Proteobacteria* present in organic (A) and mineral (B) soils. The figures depict differences in the proportions of sequences and the oligotypes.
